# Incidence and direct medical costs of child injuries in Lebanon (2012–2016): Evidence from closed insurance claims analysis

**DOI:** 10.1371/journal.pone.0353679

**Published:** 2026-07-21

**Authors:** Samar Al-Hajj, Kaitlin R. Maciejewski, Elise Presser, James Dziura, Hani Mowafi

**Affiliations:** 1 Department of Epidemiology and Biostatistics, Faculty of Health Sciences, American University of Beirut, Beirut, Lebanon; 2 Yale Center for Analytical Sciences, Yale University, New Haven, Connecticut, United States of America; 3 Department of Surgery, Yale Medical School, Yale University, New Haven, Connecticut, United States of America; 4 Department of Emergency Medicine, Yale Medical School, Yale University, New Haven, Connecticut, United States of America; Saint-Joseph University of Beirut, LEBANON

## Abstract

Despite the Eastern Mediterranean Region (EMR) reporting 20% of the global burden of child injury and one of the highest death rates for child injury (45.5 per 100,000), there are few studies from the region that analyze the costs of these conditions. This study provides an assessment of incidence and direct costs of child injury at hospitals in Lebanon using insurance closed claims. Historical claims data were compiled from the four largest Third-Party Administrators for health insurance companies (> 50% Lebanese insurance market share) for a period of 5 years from 2012–2016. Descriptive statistics and regression modeling were used to assess direct medical costs from hospital treatment of child injuries and to identify risk factors associated with increased costs. A total of 70,640 pediatric patients (<18 years) were treated for 87,102 unique injuries during 86,731 visits totaling $19,466,556.61 during the 5 year study period. Males accounted for almost 2/3 of injured children. Most children (86%) were treated and released from the emergency department but inpatient treatment accounted for over 50% of costs. The most commonly occurring injuries were minor (superficial soft tissue injuries, lacerations, open wounds). However, upper and lower extremity fractures, which accounted for only 7% of injuries, contributed to 28% of costs. Child injuries represent a significant direct medical cost burden in Lebanon, with inpatient treatment contributing a large proportion of expenes. Further prospective studies of child injury should evaluate cost-effectiveness of injury care and to identify opportunities for cost savings through standard treatment protocols for common conditions and injury prevention programs. Further studies are also needed to investigate the indirect and long-term costs of child injuries in Lebanon.

## Introduction

Injury is the leading cause of morbidity and mortality globally for children 0–17 years of age [1,2]. The 2021 Global Burden of Disease (GBD) estimated that injuries cause 4.3 million deaths, accounting for almost 8.6% of the global disease burden [3]. In addition to their large impact on child health, well-being and long-term functional impairment, injuries impose substantial economic burdens on families and health care systems, especially in resource-limited settings [4]. Unintentional injuries such as falls, road injuries, burns, drowning, and poisoning are common in children. Children’s exploratory nature, tendency to engage in risky behaviors, and underestimation of hazards in their environment place them at increased risk of sustaining unintentional injuries, some of which require unscheduled emergency care, and can lead to both short- and long- term disabilities [5].

Low- and middle-income countries (LMICs) bear sizeable injury-related costs associated with their high annual rates of injury-related morbidity and mortality [4,6]. Nevertheless, child injury estimates remain under-reported, and their costs are under-evaluated in many countries. The Eastern Mediterranean Region (EMR) reported one of highest death rates for child related unintentional injuries, estimated at 45.5 per 100,000 (global rate 38.8 per 100,000) [7]. Despite the fact that nearly 20% of the global burden of child injury takes place in the EMR [8], there remains a paucity of published literature on child injuries and their economic impacts on patients, communities and local health systems.

Existing injury-related studies in the EMR focus on specific mechanisms such as road traffic injuries (RTI) [9–14], occupational injuries [15–17], and burns [18–20]. In Lebanon, studies have highlighted costs of specific types of injuries (RTI, occupational, conflict-related) primarily in adults [12,15,21]. Child injury literature in the EMR has focused primarily on prevalence of certain injury types (e.g., school based injury) [22] or effectiveness of safety interventions (e.g., use of child safety restraints or presence of built-road safety measures to limit child RTI) [23–25]. The cost of child injuries remains a gap in the literature from the EMR [26].

The most recent comprehensive published set on health statistics in Lebanon is the *National Health Statistics Report 2018*, which compiled injury data from multiple different agency reports [27]. While this effort represented a major leap forward in terms of what is known about various injuries and health conditions in Lebanon, the aggregate report is still limited by the fragmented and non-representative data of the underlying data sets.

Lebanon currently lacks a comprehensive national child injury prevention strategy, and enforcement of child safety regulations, such as road safety measures, child restraint use, and injury prevention standards in schools and workplaces, remains limited. The absence of national injury surveillance programs and local facility-based trauma registries severely limits the ability to accurately assess the child injury burden or to estimate their associated costs to communities and the health system, which is crucial to develop evidence-based policies to reduce this pediatric burden.

While private health insurance coverage in Lebanon is low (6.5% of the population), the combination of private health insurance and employer based schemes covered roughly one-third of the population [28]. In 2017, the government, through the MOPH and military funds financed 21.4% of the cost of healthcare goods and services spent in the country (known as Current Health Expenditure, [CHE]). Funds based on employer contributions (excluding the military as an employer) such as “The National Social Security Fund” and the “Civil Servant Cooperative” covered 23%. While private insurance companies covered 19.1%, NGOs covered 3.1%. The rest are out-of-pocket (OOP) accounting for 33.1% of the healthcare expenditure (Lebanese Ministry of Public Health, 2006–2018) [29]. Furthermore, the private hospital sector is the backbone of the health care system with private hospitals attracting twice as many admissions as the public sector [28].

Knowing that pediatric injury care in Lebanon is largely delivered through hospitals, and financed mostly through private health insurance, the objective of this study is to use the high rate of private insurance penetration in Lebanon to create the first broad-based analysis of child injury in the country to provide estimates of the direct medical costs associated with hospital treatment for child injuries and to identify factors associated with higher unit cost of specific child injuries.

## Methods

### Data source

De-identified, injury-related, closed insurance claims were obtained from four leading Third Party Administrative (TPA) companies for children (<18 years) with International Classification of Diseases (ICD-10) codes corresponding to *Injury, poisoning and certain other consequences of external causes* (S00-T88) between the period of 01 January 2012–31 December 2016. TPAs manage claims data for multiple private health insurance companies in Lebanon, with each TPA handling up to 20 insurance company databases. The percentage of market share for each TPA represents the cumulative percentage of individual insurance companies’ market share managed by the TPA. Together, the TPAs included in this study represent approximately 51% of market share in Lebanon (Supplementary Table S1 in S3 Appendix).

Claims data included patient demographics, dates of service, diagnosis description, ICD code, enumerated procedures and services provided, patient disposition, National Social Security Fund Co-Insurance payment (NSSF), and total approved charges. Data on direct costs incurred at the outpatient level, in acute rehabilitation, or for care during the convalescent period at home were not available and not included in this analysis. Similarly, indirect costs were also excluded.

### Ethics declaration

The study ethical approval was obtained from the American University of Beirut (AUB) Institutional Review Board (IRB) IRB.FHS.SH.02. Informed consent was deemed unenessary to access deidentified data. This manuscript reflects original work that has not previously been published in whole or in part and is not under consideration elsewhere. The authors have read the manuscript and have agreed that the work is ready for submission and accept responsibility for its contents. The authors of this paper have complied with all ethical standards and do not have any conflicts of interest to disclose at the time of submission. All methods were performed in accordance with the ethical standards as laid down in the Declaration of Helsinki and its later amendments or comparable ethical standards.

### Data preparation

The main diagnosis description and ICD codes provided by the TPAs were found to be frequently discordant. Given that diagnosis description is most often provided by healthcare personnel, the main diagnosis description was chosen as the most likely to correctly represent the injury. In such instances of discordance, ICD 10 codes for analysis were generated based on the main diagnosis description provided by the TPAs for each individual line-item charge. For diagnosis descriptions that had multiple possible matches, the T or S code was preferentially chosen. Each charge was grouped into a service category based on the approved procedure or service provided. Service categories included *general, facility, laboratory, radiology, medication, paramedical, consultation, minor procedure, advanced procedure, surgical,* and *other*. If an approved procedure description was not included, it was classified as a *general service*. All ICD-10 reclassification and charge categorization was conducted by two clinicians with experience in trauma care (HM, EP).

### Patients and public involvement

Patients or the public were not involved in the design, or conduct, or reporting of the present study. Patients did not participate in the study in any way including defining research priority, formulating the research question, measuring outcomes, evaluating the study or disseminating the study results.

### Data analysis

This study focused on analyzing requested injury costs billed by hospitals to insurance companies ED and inpatient pediatric injury care from 01 January 2012–31 December 2016. This analysis adopts a healthcare payer/claims (third-party administrator/insurer) perspective, estimating direct medical costs captured in closed insurance claims (i.e., allowed/approved amounts recorded in the claims database). Descriptive statistics were produced to show the epidemiology of child injury in the insurance claims dataset as well as the patterns of injury.

A chi-squared test was used to identify association between gender and age, number of total visits, insurance type, National Social Security Fund Co-insurance (Co-NSSF) status, and number of injuries. Number and proportion of diagnosis code are also presented. Incidence, unadjusted mean unit cost, and total cumulative cost associated with each diagnosis code and service category were ranked. Ranks for each variable were compared to identify top contributors to overall injury cost. For each diagnosis code and service category, incidence, percent, and costs of visits were stratified by admission type (admission vs ED) and summarized.

Linear models for those treated in the Emergency Department only and those admitted for injury care were used to analyze costs of child injuries and identify factors associated with direct unit cost when controlling for age, sex, diagnosis code, and Co-NSSF status. In the regression model, data were limited to first visit per patient, line-item costs were aggregated by diagnosis code, and only diagnosis codes with at least 30 observations in ED and admit were included in the model. The cost outcome was log-transformed, given positive cost and positive skew, to appropriately model the data, and back-transformed to provide estimates. A p-value of less than 0.05 was considered statistically significant. Analyses were performed using SAS 9.4 (SAS/STATS Cary, NC).

## Results

**Fig 1:**
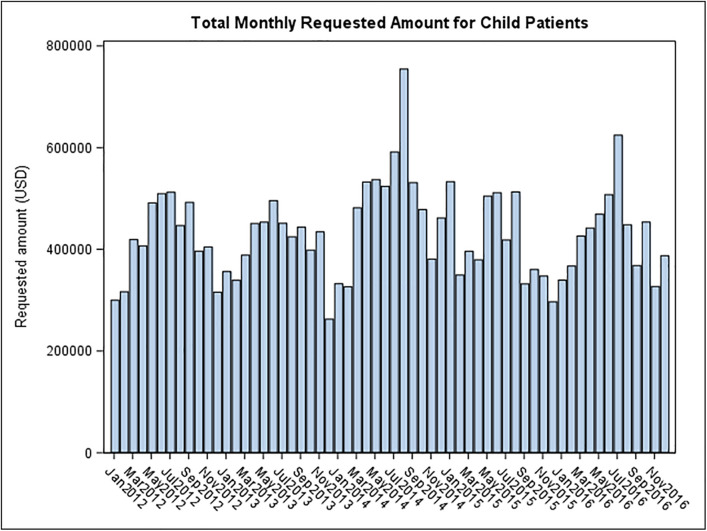
Monthly costs of child injury (January 2012 – December 2016).

Claims for a total of 87,102 injuries over 86,731 visits in 70,640 children were retrieved from leading TPA companies in Lebanon over a period of 5 years from 2012 to 2016. Fig 1 shows the total monthly cost for child injury over this period. Table 1 illustrates the characteristics of pediatric patients treated for injury. Males accounted for 2/3 of child injuries and the mean age at injury visit was 9.4 years. Almost all children (99.6%) were treated for a single injury (Table 1).

**Table 1:** Characteristics of Child Injury Patients in Lebanon.

	Female		Male		Total		
**At first visit (all unique patients)**	24,484	34.7%	46,156	65.3%	70,640	100%	P Value
**Age in years**							
Mean (SD)	8.6 (4.9)		9.8 (4.9)		9.4 (4.9)		<0.001
00–02 years	1,993	7%	2,304	4%	4,297	5%	
02–12 years	17,878	62%	30,444	53%	48,322	56%	
12–18 years	9,155	32%	24,957	43%	34,112	39%	
Mean (SD)	8.6 (4.9)		9.8 (4.9)		9.4 (4.9)		<0.001
**Number of visits**							<0.001
1	21,236	86.70%	38,527	83.5%	59,763	84.6%	
2	2,402	9.80%	5,218	11.3%	7,620	10.8%	
3+	846	3.50%	2,411	5.2%	3,257	4.6%	
**At all visits (all unique visits)**	29,026	33.5%	57,705	66.5%	86,731	100%	
**Insurance Company**							<0.001
A	5,844	20.1%	10,993	19.0%	16,837	19.4%	
B	14,343	49.4%	29,571	51.2%	43,914	50.6%	
C	7,381	25.4%	14,592	25.3%	21,973	25.3%	
D	1,458	5.0%	2,549	4.4%	4,007	4.6%	
**Co-NSSF**							< 0.001
Yes	1,843	6.3%	3,167	5.5%	5,010	5.7%	
No	27,183	93.7%	54,538	94.5%	81,721	94.2%	
**Number of injuries**							0.6
Single	28,908	99.60%	57,458	99.6%	86,366	99.6%	
Multiple	118	0.40%	247	0.4%	365	0.4%	

The incidence of each injury is shown in Table 2. The top five most common injuries include S60: Superficial injury to wrist and hand (14,132, 16.2%), S90: Superficial injury of ankle and foot (10,117, 11.6%), S01: Open wound of scalp (9,840, 11.3%), S00: Superficial injury of head (6,656, 7.6%), and S80: Superficial injury of lower leg (4,416, 5.3%). Incidence of visits were statistically different between genders.

**Table 2:** Incidence of Child Injuries by Type.

		Female		Male		Total	
**Unique diagnoses, across unique visits**		29,148	33.5%	57,954	66.5%	87,102	100%
S00	Superficial injury of head	2,408	8.3%	4,248	7.3%	6,656	7.6%
S01	Open wound of scalp	3,021	10.4%	6,819	11.8%	9,840	11.3%
S02	Fracture of skull and facial bones	162	0.6%	351	0.6%	513	0.6%
S03	Dislocation, sprain and strain of joints and ligaments of head	283	1.0%	476	0.8%	759	0.9%
S05	Injury of eye and orbit	277	1.0%	614	1.1%	891	1.0%
S06	Intracranial injury	682	2.3%	1,433	2.5%	2,115	2.4%
S09	Other and unspecified injuries of head	613	2.1%	1,456	2.5%	2,069	2.4%
S10	Superficial injury of neck	61	0.2%	101	0.2%	162	0.2%
S20	Superficial injury of thorax	157	0.5%	443	0.8%	600	0.7%
S30	Superficial injury of abdomen, lower back and pelvis	636	2.2%	1,171	2.0%	1,807	2.1%
S40	Superficial injury of shoulder and upper arm	625	2.1%	1,058	1.8%	1,683	1.9%
S42	Fracture of shoulder and upper arm	240	0.8%	386	0.7%	626	0.7%
S43	Dislocation, sprain/ strain of joints &ligaments of shoulder girdle	192	0.7%	336	0.6%	528	0.6%
S50	Superficial injury of forearm	1,393	4.8%	1,770	3.1%	3,163	3.6%
S51	Open wound of forearm	53	0.2%	189	0.3%	242	0.3%
S52	Fracture of forearm	514	1.8%	1,540	2.7%	2,054	2.4%
S53	Dislocation, sprain and strain of joints and ligaments of elbow	590	2.0%	633	1.1%	1,223	1.4%
S60	Superficial injury of wrist and hand	4,761	16.3%	9,371	16.2%	14,132	16.2%
S61	Open wound of wrist and hand	743	2.5%	2,092	3.6%	2,835	3.3%
S62	Fracture at wrist and hand level	466	1.6%	1,530	2.6%	1,996	2.3%
S63	Dislocation, sprain and strain of joints and ligaments at wrist and hand level	1,494	5.1%	3,037	5.2%	4,531	5.2%
S69	Other and unspecified injuries of wrist and hand	310	1.1%	784	1.4%	1,094	1.3%
S70	Superficial injury of hip and thigh	187	0.6%	412	0.7%	599	0.7%
S72	Fracture of femur	63	0.2%	185	0.3%	248	0.3%
S73	Dislocation, sprain and strain of joint and ligaments of hip	105	0.4%	181	0.3%	286	0.3%
S80	Superficial injury of lower leg	1,536	5.3%	3,080	5.3%	4,616	5.3%
S81	Open wound of lower leg	204	0.7%	589	1.0%	793	0.9%
S82	Fracture of lower leg, including ankle	189	0.6%	521	0.9%	710	0.8%
S83	Dislocation, sprain and strain of joints and ligaments of knee	454	1.6%	929	1.6%	1,383	1.6%
S90	Superficial injury of ankle and foot	3,657	12.5%	6,460	11.1%	10,117	11.6%
S91	Open wound of ankle and foot	391	1.3%	821	1.4%	1,212	1.4%
S92	Fracture of foot, except ankle	120	0.4%	361	0.6%	481	0.6%
S93	Dislocation, sprain and strain of joints and ligaments at ankle and foot level	1,362	4.7%	2,480	4.3%	3,842	4.4%
T00	Superficial injuries involving multiple body regions	170	0.6%	378	0.7%	548	0.6%
T07	Unspecified multiple injuries	65	0.2%	161	0.3%	226	0.3%
T14	Injury of unspecified body region	362	1.2%	763	1.3%	1,125	1.3%
T17	Foreign body in respiratory tract	243	0.8%	296	0.5%	539	0.6%
T18	Foreign body in alimentary tract	174	0.6%	228	0.4%	402	0.5%
T50	Poisoning by, adverse effect of and underdosing of diuretics and other and unspecified drugs, medicaments and biological substances	48	0.2%	55	0.1%	103	0.1%
T75	Effects of other external causes	21	0.1%	58	0.1%	79	0.1%
T78	Adverse effects, not elsewhere classified	116	0.4%	158	0.3%	274	0.3%

**Fig 2:**
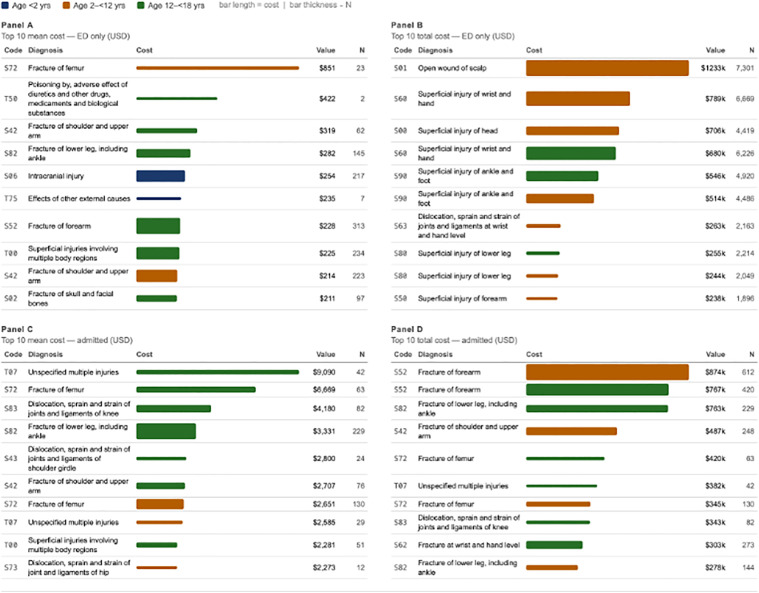
Top 10 Child Injury Costs ED vs Admitted.

Fig 2 shows the top 10 injuries with the highest mean and total costs by patient disposition with overall cost during this period totalling $19,466,557. While 86% of injuries were treated and discharged from the ED, the 14% admitted contributed 44% of overall child injury cost. For patients treated in the ED and discharged mean cost (Panel A) is dominated by fractures and intracranial injuries with modest N, while total cost (Panel B) is almost entirely due to high-volume superficial injuries – primarily of the scalp in very young children. For children requiring admission for inpatient injury care, both mean cost (Panel C) and total cost (Panel D) are due to fractures, other orthopedic injuries (dislocation and sprain), and *unspecified multiple injuries* category accounting for the highest mean cost per admission ($9,090). Additional detail available in Table S4 in S3 Appendix. *Rank, Mean cost, and Total Cost of Child Injury in Lebnon – ED, Admit, and Total.*

Table 3 shows the categorization of individual line item charges for services. A single lump-sum “General” service charge accounted for 75.9% of charges. Itemized charges were primarily for minor procedures (9.4%) such as bedside laceration repair; Advanced procedures (4.8%) including those that required specialized equipment or procedural suites such as endoscopy or interventional radiology procedures; and the remaining 10% of billed items for facility charges, physician consultation, and diagnostic studies.

**Table 3:** Categories of Child Injury Cost by Admission Type for Insured Patients in Lebanon.

Cost Category	Emergency Dept Charges				Hospital Inpatient Charges				Total Charges			
	N	Charges (%)	Cost by Category ($)	Cost (%)	N	Charges (%)	Costs by Category ($)	Cost (%)	N	Charges (%)	Cost ($)	Cost (%)
Advanced Procedure	3,471	4.0	578,151	5.3	940	7.0	885,329	10.3	4,411	4.8	1,463,479	7.5
Consultation(Physician)	349	0.0	18,825	0.2	1,871	15.0	98,620	1.1	2,220	2.4	117,445	0.6
Facility costs	434	1.0	15,280	0.1	1,971	16.0	106,996	1.2	2,405	2.6	122,276	0.6
General (Not otherwise specified)	66,715	83.0	8,593,543	79.1	3,496	28.0	4,371,270	50.8	70,211	75.9	1,964,813	66.6
Laboratory studies	19	0.0	939	0.0	151	1.0	8,152	0.1	170	0.2	9,091	0.0
Medications	128	0.0	1,359	0.0	540	4.0	10,334	0.1	668	0.7	11,693	0.1
Other Diagnostic Tests	4	0.0	313	0.0	8	0.0	5,646	0.1	12	0.0	5,960	0.0
Paramedical Professional Services	144	0.0	2,412	0.0	443	4.0	30,948	0.4	587	0.6	33,361	0.2
Procedure (Minor)	7,911	10.0	1,573,639	14.5	749	6.0	261,078	3.0	8,660	9.4	1,834,717	9.4
Diagnostic Imaging	525	1.0	27,256	0.3	1,414	11.0	75,360	0.9	1,939	2.1	102,615	0.5
Surgical	224	0.0	49,660	0.5	1,003	8.0	2,751,448	32.0	1,227	1.3	2,801,108	14.4
**Total**	79,924	100.0	10,861,376	100.0	12,586	100.0	8,605,181	100.0	92,510	100.0	19,466,557	100.0

*Costs are rounded to the nearest whole dollar. Percentages are reported to one decimal place.

Separate regression models for ED patients (S2 Table in S3 Appendix) and Admitted patients (S3 Table in S3 Appendix) revealed that emergency department injury treatment costs varied only by age with no significant differences between sex, or by Co-NSSF insurance status. This is consistent with billing of most presentations with a single global general service charge. Inpatient injury costs varied by age as well as diagnosis with geometric mean costs for orthopedic injuries (primarily extremity fractures or dislocations) being 3x – 7x cost of other injuries treated (Supplementary Tables S2 & S3 in S3 Appendix – models; and Table S4 in S3 Appendix – actual ED and hospital incidence, mean and total costs by diagnosis).

## Discussion

This study presents the first national insurance-claims-based estimates of the direct costs of child injury related to Emergency Department (ED) and hospital inpatient treatment over a period of five years in Lebanon. The study provides an initial estimate of the economic burden of such injuries while also providing a baseline to assess the cost effectiveness of future injury prevention programs.

Consistent with existing evidence, this study found that males were more likely to experience injury in childhood [30–35]. While over 86% of injuries were treated and discharged from the ED, inpatient treatment drove most of the overall cost. Unit cost of emergency department injury treatment was lower and contribution of ED based care to overall costs was related to the volume of cases. Inpatient unit costs for each diagnosis codes were higher (often by an order of magnitude) and their contributions to overall cost were from a combination of incidence and unit cost. Notably, extremity fractures accounted for 28% of total costs while only accounting for 7% of injuries. These findings suggest that resource allocation and cost reduction efforts should focus on high-cost injury types and settings, and highlight the potential for targeted strategies to improve efficiency in pediatric injury care.

While these analyses were not designed to quantify the total cost of child injuries, and importantly they leave out costs associated with outpatient and rehabilitative care, as well as the indirect costs associated with lost school and work, this paper does provide insight into the substantial economic burden of child injury on the local healthcare system.

Lebanon represents a unique case in the Eastern Mediterranean region with its relative high penetration of private medical insurance that covers a plurality of Lebanese residents (46.8% - private or employer based health insurance; 45.6% - government health insurance; 7.6% - self-paid or UN/international agencies) [35].

This study’s average child injury direct cost of $224.44 per injury is consistent with the average injury direct cost of $291 per injury mechanism found in a recent economic review of injury in LMICs [4]. In that study, Wesson et al. also emphasized the “lack of injury-related economic evidence from LMICs” and the “considerable variability in costs and cost-descriptions” suggesting that while economic burden of injury is high, the generalizability of studies within their review was limited. Other research has shown that the total cost of injury may be up to 14 times the direct cost when accounting for indirect injury cost and direct non-medical costs [4]. These estimates show the large potential financial impact of injury on individuals, families, and local healthcare systems.

This study underlines the urgent need to adopt child injury prevention strategies and cost-effective interventions in Lebanon, a country that lacks significant prevention programs and safety policies, to reduce child injury frequency and severity. Ample injury prevention programs and policies, proven successful in lowering the frequency and magnitude of injuries worldwide, can be contextualized and implemented to reduce the child injury burden in Lebanon [34,36–40]. Successful strategies include school- and community-based educational interventions, environmental modifications, and enforcement of safety regulations, particularly in road traffic, recreational, and sports settings. Specifically, modeling studies in LMICs show that targeted interventions, such as traffic enforcement, speed bumps, helmet promotion, and safe storage of hazardous substances, can be highly cost-effective, with enhanced traffic enforcement alone averaged $64 per DALY averted [34].

This study has multiple strengths. First, this study accessed a large, administrative, closed claims database covering over 50% of the insurance national market share in Lebanon. As such, the data reports a widely diverse pool of beneficiaries that span geographically dispersed locations across Lebanon and belong to different economic statuses.

This study has some limitations. First, this study relies solely on secondary data accessed from insurance claims database, hence data analysis and outcomes is limited to the existing variables collected in the insurance’s administrative database. Importantly, these injury claims lack any information related to the mechanism of injury. Second, the study encompasses only the direct costs of child injuries without accounting for the indirect costs of injuries including physical property damage, loss of productivity, patients’ pain and suffering, patients’ DALYs, house modifications, and caregiver absenteeism (i.e., parents missing work days to care for their children), all of which contribute to the overall cost of an injury.

In addition, the data underlying this analysis are now ten years old. This reflects (i) the substantial time required to obtain approvals from multiple independent insurance stakeholders and (ii) stakeholders’ preference to share historical closed claims rather than more recent claims that could still be subject to appeal or other administrative processes. Importantly, the selected period also corresponds to exceptionally low inflation and relative price stability in Lebanon, improving internal comparability of cost estimates across years and offering a valuable pre-crisis baseline against which post-2019 patterns may be interpreted in future work, given the hyperinflationary context after 2019.

## Conclusion

Child injury is a major health problem with significant direct costs to the health system in Lebanon. The results found in this study show the relative high cost of orthopedic care in comparison to other injury care provided for child injuries in Lebanon. These findings highlight the importance of targeting high-cost injuries for prevention and exploring more cost-effective treatment strategies. While this study focuses on insurer-reimbursed direct costs, further research is needed to capture indirect and long-term costs and to guide comprehensive, cost-informed pediatric injury prevention policies in Lebanon.

## Supporting information

S1 Appendix Appendix A: Adjusted mean direct cost for injured children treated in the Emergency Department alone *(controlling for age, gender, co-NSSF status, and diagnosis code).* (PDF)

S2 Appendix Appendix B: Adjusted mean direct cost for injured children admitted to the hospital for treatment *(controlling for age, gender, co-NSSF status, and diagnosis code).* (PDF)

S3 Appendix Appendix C: Supplementary Tables: Table S1, Table S2, Table S3, Table S4. (DOCX)
