# Supplementary material for: Incidence and direct medical costs of child injuries in Lebanon (2012–2016): Evidence from closed insurance claims analysis
Source: PLoS One. 2026 Jul 21;21(7):e0353679. doi: 10.1371/journal.pone.0353679 (PMC13387535; doi:10.1371/journal.pone.0353679)
Supplement: S1 Appendix — (PDF) [file pone.0353679.s001.pdf]

Appendix A: Adjusted mean direct cost for injured children treated in the Emergency Department alone
(controlling for age, gender, co-NSSF status, and diagnosis code)

		Mean (USD)	95% CI		Type-3 P-value
**Age (years)**		$ 0.99	$ 0.99	$ 1.00	<0.001
**Gender**					0.2
Female		$119.52	$118.04	$121.04	
Male		$120.19	$118.80	$121.61	
**Co-NSSF**					0.18
Yes		$120.65	$118.31	$123.04	
No		$119.07	$118.19	$119.96	
**Diagnosis code**					
S00	Superficial injury of head	$125.42	$123.33	$127.55	
S01	Open wound of scalp	$151.93	$149.70	$154.19	
S02	Fracture of skull and facial bones	$114.66	$107.35	$122.47	
S03	Dislocation, sprain and strain of joints and ligaments of head	$123.17	$118.13	$128.43	
S05	Injury of eye and orbit	$123.54	$118.82	$128.44	
S06	Intracranial injury	$142.79	$138.89	$146.80	
S09	Other and unspecified injuries of head	$132.71	$129.19	$136.35	
S30	Superficial injury of abdomen, lower back and pelvis	$117.46	$114.16	$120.84	
S40	Superficial injury of shoulder and upper arm	$122.56	$118.97	$126.24	
S42	Fracture of shoulder and upper arm	$140.06	$131.18	$149.53	
S50	Superficial injury of forearm	$106.40	$104.02	$108.83	
S52	Fracture of forearm	$141.03	$135.98	$146.28	
S53	Dislocation, sprain and strain of joints and ligaments of elbow	$108.51	$104.73	$112.43	
S60	Superficial injury of wrist and hand	$101.08	$99.76	$102.41	
S61	Open wound of wrist and hand	$142.49	$139.23	$145.84	
S62	Fracture at wrist and hand level	$119.47	$115.95	$123.10	
S63	Dislocation, sprain and strain of joints and ligaments at wrist and hand level	$102.23	$100.26	$104.23	
S69	Other and unspecified injuries of wrist and hand	$99.58	$96.10	$103.20	
S70	Superficial injury of hip and thigh	$108.70	$103.66	$114.00	
S80	Superficial injury of lower leg	$103.68	$101.70	$105.70	
S81	Open wound of lower leg	$154.95	$148.49	$161.69	
S82	Fracture of lower leg, including ankle	$136.29	$128.18	$144.91	
S83	Dislocation, sprain and strain of joints and ligaments of knee	$105.37	$101.99	$108.85	
S90	Superficial injury of ankle and foot	$101.77	$100.30	$103.25	
S91	Open wound of ankle and foot	$141.17	$136.41	$146.12	
S92	Fracture of foot, except ankle	$116.71	$110.32	$123.46	
S93	Dislocation, sprain and strain of joints and ligaments at ankle and foot level	$101.04	$98.97	$103.16	
T00	Superficial injuries involving multiple body regions	$165.75	$157.01	$175.00	
T07	Unspecified multiple injuries	$131.80	$121.09	$143.45	
T14	Injury of unspecified body region	$143.17	$137.92	$148.61	
T17	Foreign body in respiratory tract	$100.95	$95.81	$106.36	
T18	Foreign body in alimentary tract	$101.44	$95.48	$107.79	
T78	Adverse effects, not elsewhere classified	$80.05	$74.31	$86.22	
