# Supplementary material for: Incidence and direct medical costs of child injuries in Lebanon (2012–2016): Evidence from closed insurance claims analysis
Source: PLoS One. 2026 Jul 21;21(7):e0353679. doi: 10.1371/journal.pone.0353679 (PMC13387535; doi:10.1371/journal.pone.0353679)
Supplement: S2 Appendix — (PDF) [file pone.0353679.s002.pdf]

Appendix B: Adjusted mean direct cost for injured children admitted to the hospital for treatment
(controlling for age, gender, co-NSSF status, and diagnosis code)

		Mean (USD)	95% CI		Type-3 P-value
**Age (years)**		$ 1.01	$ 1.00	$ 1.01	0.02
**Gender**					0.11
Female	Female	$358.02	$338.02	$379.21	
Male	Male	$375.14	$358.10	$393.04	
**Co-NSSF**					0.3
Yes	Yes	$372.56	$349.78	$396.83	
No	No	$360.50	$345.54	$376.08	
**Diagnosis code**					
S00	Superficial injury of head	$140.16	$126.58	$155.21	
S01	Open wound of scalp	$167.15	$151.85	$184.01	
S02	Fracture of skull and facial bones	$789.34	$677.22	$920.12	
S03	Dislocation, sprain and strain of joints and ligaments of head	$175.77	$125.34	$246.51	
S05	Injury of eye and orbit	$386.18	$292.83	$509.28	
S06	Intracranial injury	$638.93	$575.71	$709.10	
S09	Other and unspecified injuries of head	$450.97	$376.53	$540.12	
S30	Superficial injury of abdomen, lower back and pelvis	$138.53	$110.74	$173.28	
S40	Superficial injury of shoulder and upper arm	$108.30	$84.12	$139.42	
S42	Fracture of shoulder and upper arm	$1,679.41	$1,478.52	$1,907.60	
S50	Superficial injury of forearm	$129.48	$108.96	$153.85	
S52	Fracture of forearm	$1,178.38	$1,093.24	$1,270.16	
S53	Dislocation, sprain and strain of joints and ligaments of elbow	$261.73	$211.09	$324.50	
S60	Superficial injury of wrist and hand	$80.30	$74.19	$86.93	
S61	Open wound of wrist and hand	$329.18	$281.13	$385.45	
S62	Fracture at wrist and hand level	$626.34	$559.48	$701.20	
S63	Dislocation, sprain and strain of joints and ligaments at wrist and hand level	$153.35	$122.97	$191.23	
S69	Other and unspecified injuries of wrist and hand	$783.76	$582.60	$1,054.48	
S70	Superficial injury of hip and thigh	$201.97	$137.21	$297.32	
S80	Superficial injury of lower leg	$86.32	$74.93	$99.44	
S81	Open wound of lower leg	$272.84	$204.00	$364.93	
S82	Fracture of lower leg, including ankle	$1,653.91	$1,471.44	$1,859.01	
S83	Dislocation, sprain and strain of joints and ligaments of knee	$2,364.68	$1,863.48	$3,001.00	
S90	Superficial injury of ankle and foot	$82.67	$74.42	$91.84	
S91	Open wound of ankle and foot	$220.19	$162.85	$297.73	
S92	Fracture of foot, except ankle	$741.52	$559.20	$983.19	
S93	Dislocation, sprain and strain of joints and ligaments at ankle and foot level	$173.82	$135.86	$222.38	
T00	Superficial injuries involving multiple body regions	$783.13	$622.41	$985.35	
T07	Unspecified multiple injuries	$1,341.04	$1,037.22	$1,733.85	
T14	Injury of unspecified body region	$513.53	$433.50	$608.32	
T17	Foreign body in respiratory tract	$693.12	$531.39	$904.15	
T18	Foreign body in alimentary tract	$637.78	$482.22	$843.53	
T78	Adverse effects, not elsewhere classified	$545.33	$377.02	$788.79	
