# Supplementary material for: Incidence and direct medical costs of child injuries in Lebanon (2012–2016): Evidence from closed insurance claims analysis
Source: PLoS One. 2026 Jul 21;21(7):e0353679. doi: 10.1371/journal.pone.0353679 (PMC13387535; doi:10.1371/journal.pone.0353679)
Supplement: S3 Appendix — (DOCX) [file pone.0353679.s003.docx]

**Appendix C**

**Table S1: Child Injury ED Cost, Admit Cost, Total Cost by Diagnosis**

|  | | **Emergency costs by diagnosis code** | | | | | |  | **Admitted costs by diagnosed code** | | | | | |  | **Overall costs by diagnosis code** | | | | | |
| --- | --- | --- | --- | --- | --- | --- | --- | --- | --- | --- | --- | --- | --- | --- | --- | --- | --- | --- | --- | --- | --- |
| **Diagnosis code category** | | **Observation / Injury** | | **Mean Unit Cost** | | **ED Total Cost** | |  | **Observation / Injury** | | **Mean Unit Cost** | | **Admit Total Cost** | |  | **Observation / Injury** | | **Mean Unit Cost** | | **Overall Total Cost** | |
|  | | **Rank** | **N** | **Rank** | **Unit ($)** | **Rank** | **Total ($)** |  | **Rank** | **N** | **Rank** | **Unit ($)** | **Rank** | **Total ($)** |  | **Rank** | **N** | **Rank** | **Unit ($)** | **Rank** | **Total ($)** |
| S52 | Fracture of forearm | 19 | 1,013 | 5 | 200 | 15 | 202,742 |  | 1 | 1,041 | 9 | 1,593 | 1 | 1,658,490 |  | 12 | 2,054 | 9 | 1,593 | 1 | 1,861,232 |
| S01 | Open wound of scalp | 3 | 9,232 | 11 | 170 | 1 | 1,565,213 |  | 3 | 608 | 31 | 300 | 11 | 182,300 |  | 3 | 9,840 | 31 | 300 | 2 | 1,747,513 |
| S60 | Superficial injury of wrist and hand | 1 | 13,167 | 37 | 114 | 2 | 1,503,923 |  | 2 | 965 | 40 | 139 | 16 | 133,645 |  | 1 | 14,132 | 40 | 139 | 3 | 1,637,568 |
| S90 | Superficial injury of ankle and foot | 2 | 9,572 | 39 | 113 | 3 | 1,083,490 |  | 4 | 545 | 39 | 141 | 20 | 76,952 |  | 2 | 10,117 | 39 | 141 | 4 | 1,160,442 |
| S82 | Fracture of lower leg, including ankle | 31 | 327 | 3 | 221 | 27 | 72,263 |  | 8 | 383 | 4 | 2,759 | 2 | 1,056,584 |  | 24 | 710 | 4 | 2,759 | 5 | 1,128,846 |
| S00 | Superficialinjury of head | 4 | 6,131 | 13 | 160 | 4 | 979,089 |  | 5 | 525 | 35 | 256 | 15 | 134,502 |  | 4 | 6,656 | 35 | 256 | 6 | 1,113,592 |
| S06 | Intracranial injury | 12 | 1,648 | 6 | 196 | 10 | 323,611 |  | 6 | 467 | 15 | 1,187 | 5 | 554,400 |  | 10 | 2,115 | 15 | 1,187 | 7 | 878,010 |
| S72 | Fracture of femur | 41 | 36 | 1 | 608 | 37 | 21,879 |  | 12 | 212 | 3 | 3,785 | 3 | 802,458 |  | 36 | 248 | 3 | 3,785 | 8 | 824,337 |
| S42 | Fracture of shoulder and upper arm | 32 | 297 | 2 | 233 | 29 | 69,146 |  | 9 | 329 | 5 | 2,137 | 4 | 702,906 |  | 25 | 626 | 5 | 2,137 | 9 | 772,052 |
| S62 | Fracture at wrist and hand level | 14 | 1,559 | 20 | 149 | 13 | 231,690 |  | 7 | 437 | 18 | 1,085 | 6 | 474,088 |  | 13 | 1,996 | 18 | 1,085 | 10 | 705,778 |
| S61 | Open wound of wrist and hand | 9 | 2,630 | 14 | 157 | 8 | 413,783 |  | 14 | 205 | 24 | 698 | 13 | 143,083 |  | 9 | 2,835 | 24 | 698 | 11 | 556,867 |
| S63 | Dislocation,sprain and strain of joints and ligaments at wrist and hand level | 5 | 4,426 | 35 | 116 | 5 | 513,441 |  | 19 | 105 | 30 | 346 | 31 | 36,305 |  | 6 | 4,531 | 30 | 346 | 12 | 549,746 |
| S80 | Superficial injury of lower leg | 6 | 4,327 | 34 | 118 | 6 | 508,259 |  | 10 | 289 | 41 | 127 | 30 | 36,794 |  | 5 | 4,616 | 41 | 127 | 13 | 545,053 |
| S83 | Dislocation,sprain and strain of joints and ligaments of knee | 15 | 1,291 | 23 | 136 | 18 | 175,792 |  | 20 | 92 | 2 | 3,912 | 8 | 359,879 |  | 16 | 1,383 | 2 | 3,912 | 14 | 535,670 |
| T07 | Unspecified multiple injuries | 37 | 152 | 8 | 177 | 35 | 26,825 |  | 24 | 74 | 1 | 6,201 | 7 | 458,840 |  | 38 | 226 | 1 | 6,201 | 15 | 485,665 |
| S93 | Dislocation, sprain and strain of joints and ligaments at ankle and foot level | 7 | 3,758 | 36 | 115 | 7 | 430,529 |  | 22 | 84 | 26 | 600 | 26 | 50,424 |  | 7 | 3,842 | 26 | 600 | 16 | 480,953 |
| S09 | Other and unspecified injuries of head | 10 | 1,921 | 15 | 157 | 11 | 302,119 |  | 16 | 148 | 20 | 934 | 14 | 138,289 |  | 11 | 2,069 | 20 | 934 | 17 | 440,407 |
| S50 | Superficial injury of forearm | 8 | 2,953 | 33 | 122 | 9 | 359,301 |  | 13 | 210 | 34 | 271 | 25 | 56,963 |  | 8 | 3,163 | 34 | 271 | 18 | 416,264 |
| S02 | Fracture of skull and facial bones | 33 | 292 | 9 | 172 | 32 | 50,283 |  | 11 | 221 | 10 | 1,591 | 9 | 351,547 |  | 31 | 513 | 10 | 1,591 | 19 | 401,830 |
| T14 | Injury of unspecified body region | 20 | 959 | 7 | 184 | 17 | 176,848 |  | 15 | 166 | 17 | 1,097 | 12 | 182,051 |  | 19 | 1,125 | 17 | 1,097 | 20 | 358,898 |
| T00 | Superficial injuries involving multiple body regions | 28 | 458 | 4 | 207 | 24 | 94,559 |  | 21 | 90 | 6 | 2,108 | 10 | 189,678 |  | 28 | 548 | 6 | 2,108 | 21 | 284,236 |
| S30 | Superficial injury of abdomen, lower back and pelvis | 11 | 1,699 | 24 | 136 | 14 | 231,058 |  | 18 | 108 | 36 | 237 | 34 | 25,620 |  | 14 | 1,807 | 36 | 237 | 22 | 256,678 |
| S40 | Superficial injury of shoulder and upper arm | 13 | 1,599 | 21 | 145 | 12 | 231,696 |  | 22 | 84 | 37 | 166 | 37 | 13,966 |  | 15 | 1,683 | 37 | 166 | 23 | 245,662 |
| S53 | Dislocation,sprain and strain of joints and ligaments of elbow | 17 | 1,114 | 28 | 127 | 19 | 141,067 |  | 17 | 109 | 23 | 725 | 19 | 78,990 |  | 17 | 1,223 | 23 | 725 | 24 | 220,056 |
| S91 | Open wound of ankle and foot | 16 | 1,151 | 17 | 156 | 16 | 179,185 |  | 29 | 61 | 29 | 509 | 32 | 31,059 |  | 18 | 1,212 | 29 | 509 | 25 | 210,244 |
| S05 | Injury of eye and orbit | 21 | 822 | 18 | 155 | 20 | 127,012 |  | 26 | 69 | 14 | 1,193 | 18 | 82,316 |  | 21 | 891 | 14 | 1,193 | 26 | 209,327 |
| S69 | Other and unspecified injuries of wrist and hand | 18 | 1,037 | 38 | 114 | 22 | 118,104 |  | 31 | 57 | 12 | 1,252 | 21 | 71,335 |  | 20 | 1,094 | 12 | 1,252 | 27 | 189,439 |
| S81 | Open wound of lower leg | 22 | 725 | 10 | 170 | 21 | 122,971 |  | 27 | 68 | 25 | 614 | 27 | 41,752 |  | 22 | 793 | 25 | 614 | 28 | 164,724 |
| S92 | Fracture of foot, except ankle | 29 | 414 | 29 | 127 | 31 | 52,395 |  | 28 | 67 | 11 | 1,375 | 17 | 92,125 |  | 32 | 481 | 11 | 1,375 | 29 | 144,520 |
| S43 | Dislocation, sprain and strain of joints and ligaments of shoulder girdle | 26 | 494 | 19 | 151 | 26 | 74,659 |  | 34 | 34 | 7 | 2,034 | 22 | 69,153 |  | 30 | 528 | 7 | 2,034 | 30 | 143,811 |
| T17 | Foreign body in respiratory tract | 27 | 469 | 32 | 123 | 30 | 57,844 |  | 25 | 70 | 19 | 978 | 23 | 68,443 |  | 29 | 539 | 19 | 978 | 31 | 126,287 |
| S03 | Dislocation, sprain and strain of joints and ligaments of head | 23 | 716 | 16 | 156 | 23 | 111,529 |  | 32 | 43 | 32 | 289 | 39 | 12,437 |  | 23 | 759 | 32 | 289 | 32 | 123,966 |
| S70 | Superficial injury of hip and thigh | 25 | 565 | 27 | 127 | 28 | 71,564 |  | 34 | 34 | 13 | 1,221 | 28 | 41,495 |  | 27 | 599 | 13 | 1,221 | 33 | 113,059 |
| T18 | Foreign body in alimentary tract | 30 | 341 | 31 | 124 | 33 | 42,139 |  | 29 | 61 | 16 | 1,119 | 24 | 68,253 |  | 33 | 402 | 16 | 1,119 | 34 | 110,392 |
| S20 | Superficial injury of thorax | 24 | 570 | 25 | 132 | 25 | 75,439 |  | 36 | 30 | 33 | 278 | 40 | 8,329 |  | 26 | 600 | 33 | 278 | 35 | 83,768 |
| S73 | Dislocation, sprain and strain of joint and ligaments of hip | 34 | 263 | 40 | 95 | 36 | 25,033 |  | 40 | 23 | 8 | 1,734 | 29 | 39,889 |  | 34 | 286 | 8 | 1,734 | 36 | 64,922 |
| S51 | Open wound of forearm | 36 | 216 | 12 | 167 | 34 | 36,167 |  | 39 | 26 | 27 | 525 | 38 | 13,659 |  | 37 | 242 | 27 | 525 | 37 | 49,826 |
| T78 | Adverse effects, not elsewhere classified | 35 | 239 | 41 | 91 | 38 | 21,797 |  | 33 | 35 | 22 | 768 | 33 | 26,868 |  | 35 | 274 | 22 | 768 | 38 | 48,665 |
| T75 | Effects of other external causes | 40 | 51 | 26 | 128 | 41 | 6,512 |  | 37 | 28 | 21 | 792 | 35 | 22,162 |  | 41 | 79 | 21 | 792 | 39 | 28,674 |
| T50 | Burn and corrosion, body region unspecified | 39 | 75 | 30 | 126 | 40 | 9,478 |  | 37 | 28 | 28 | 514 | 36 | 14,398 |  | 40 | 103 | 28 | 514 | 40 | 23,876 |
| S10 | Superficial injury of neck | 38 | 145 | 22 | 144 | 39 | 20,944 |  | 41 | 17 | 38 | 162 | 41 | 2,758 |  | 39 | 162 | 38 | 162 | 41 | 23,701 |

**Table S2: Linear regression model for cost of Emergency Department based child injury care in private Lebanese hospitals (USD) *(controlling for age, gender, co-NSSF status, and diagnosis code)***

| **Age (Years)** | |  | **Mean (USD)** | **95% CI** | | **Type-3  P-value** |
| --- | --- | --- | --- | --- | --- | --- |
|  | 0 to < 2 |  | $ 121.13 | $ 118.65 | $ 123.68 | <0.001 |
|  | 2 to < 12 |  | $ 115.55 | $ 113.95 | $ 117.17 |  |
|  | 12 to <18 years |  | $ 118.00 | $ 116.47 | $ 119.56 |  |
| **Gender** | |  |  |  |  |  |
|  | Female |  | $ 118.08 | $ 116.40 | $ 119.79 | 0.62 |
|  | Male |  | $ 118.33 | $ 116.71 | $ 119.98 |  |
| **Co-NSSF** | |  |  |  |  |  |
|  | **No** |  | $ 117.78 | $ 116.51 | $ 119.06 | 0.44 |
|  | **Yes** |  | $ 118.64 | $ 116.26 | $ 121.06 |  |
| **Diagnosis code** | |  |  |  |  |  |
|  | S00 | Superficial injury of head | $ 128.03 | $ 125.90 | $ 130.22 |  |
|  | S01 | Open wound of scalp | $ 154.93 | $ 152.64 | $ 157.28 |  |
|  | S02 | Fracture of skull and facial bones | $ 114.65 | $ 107.30 | $ 122.51 |  |
|  | S03 | Dislocation, sprain and strain of joints and ligaments of head | $ 124.36 | $ 119.19 | $ 129.76 |  |
|  | S05 | Injury of eye and orbit | $ 126.09 | $ 121.26 | $ 131.12 |  |
|  | S06 | Intracranial injury | $ 144.33 | $ 140.32 | $ 148.46 |  |
|  | S09 | Other and unspecified injuries of head | $ 134.77 | $ 131.14 | $ 138.50 |  |
|  | S10 | Superficial injury of neck | $ 121.94 | $ 111.36 | $ 133.53 |  |
|  | S20 | Superficial injury of thorax | $ 117.65 | $ 112.11 | $ 123.46 |  |
|  | S30 | Superficial injury of abdomen, lower back and pelvis | $ 117.45 | $ 114.07 | $ 120.92 |  |
|  | S40 | Superficial injury of shoulder and upper arm | $ 123.25 | $ 119.61 | $ 127.00 |  |
|  | S42 | Fracture of shoulder and upper arm | $ 138.86 | $ 130.15 | $ 148.16 |  |
|  | S43 | Dislocation, sprain and strain of joints and ligaments of shoulder girdle | $ 128.38 | $ 121.95 | $ 135.17 |  |
|  | S50 | Superficial injury of forearm | $ 107.57 | $ 105.12 | $ 110.07 |  |
|  | S51 | Open wound of forearm | $ 150.78 | $ 139.45 | $ 163.02 |  |
|  | S52 | Fracture of forearm | $ 142.78 | $ 137.59 | $ 148.18 |  |
|  | S53 | Dislocation, sprain and strain of joints and ligaments of elbow | $ 109.49 | $ 105.67 | $ 113.45 |  |
|  | S60 | Superficial injury of wrist and hand | $ 101.45 | $ 100.05 | $ 102.87 |  |
|  | S61 | Open wound of wrist and hand | $ 142.78 | $ 139.44 | $ 146.20 |  |
|  | S62 | Fracture at wrist and hand level | $ 119.83 | $ 116.26 | $ 123.53 |  |
|  | S63 | Dislocation, sprain and strain of joints and ligaments at wrist and hand level | $ 102.45 | $ 100.41 | $ 104.54 |  |
|  | S69 | Other and unspecified injuries of wrist and hand | $ 99.54 | $ 96.00 | $ 103.23 |  |
|  | S70 | Superficial injury of hip and thigh | $ 109.75 | $ 104.61 | $ 115.15 |  |
|  | S72 | Fracture of femur | $ 153.71 | $ 128.18 | $ 184.36 |  |
|  | S73 | Dislocation, sprain and strain of joint and ligaments of hip | $ 43.40 | $ 40.58 | $ 46.41 |  |
|  | S80 | Superficial injury of lower leg | $ 103.93 | $ 101.87 | $ 106.02 |  |
|  | S81 | Open wound of lower leg | $ 155.23 | $ 148.71 | $ 162.03 |  |
|  | S82 | Fracture of lower leg, including ankle | $ 136.40 | $ 128.25 | $ 145.08 |  |
|  | S83 | Dislocation, sprain and strain of joints and ligaments of knee | $ 105.57 | $ 102.10 | $ 109.16 |  |
|  | S90 | Superficial injury of ankle and foot | $ 101.88 | $ 100.33 | $ 103.45 |  |
|  | S91 | Open wound of ankle and foot | $ 141.63 | $ 136.77 | $ 146.64 |  |
|  | S92 | Fracture of foot, except ankle | $ 116.83 | $ 110.35 | $ 123.68 |  |
|  | S93 | Dislocation, sprain and strain of joints and ligaments at ankle and foot level | $ 101.15 | $ 99.00 | $ 103.34 |  |
|  | T00 | Superficial injuries involving multiple body regions | $ 167.12 | $ 158.24 | $ 176.50 |  |
|  | T07 | Unspecified multiple injuries | $ 137.02 | $ 126.10 | $ 148.87 |  |
|  | T14 | Injury of unspecified body region | $ 144.36 | $ 138.98 | $ 149.95 |  |
|  | T17 | Foreign body in respiratory tract | $ 103.19 | $ 97.91 | $ 108.77 |  |
|  | T18 | Foreign body in alimentary tract | $ 104.31 | $ 98.15 | $ 110.86 |  |
|  | T50 | Poisoning by, adverse effect of and underdosing of diuretics and other and unspecified drugs, medicaments and biological substances | $ 97.59 | $ 85.89 | $ 110.89 |  |
|  | T75 | Effects of other external causes | $ 100.86 | $ 85.71 | $ 118.66 |  |
|  | T78 | Adverse effects, not elsewhere classified | $ 81.32 | $ 75.49 | $ 87.59 |  |

**Table S3: Linear regression model for cost of hospital inpatient child injury care in private Lebanese hospitals (USD) *(controlling for age, gender, co-NSSF status, and diagnosis code)***

| **Age (Years)** | | | **Mean (USD)** | **95% CI** | | **Type-3  P-value** |
| --- | --- | --- | --- | --- | --- | --- |
|  | 0 to < 2 | | $ 407.52 | $ 365.66 | $ 454.23 | <0.001 |
|  | 2 to < 12 | | $ 345.95 | $ 331.99 | $ 360.50 |  |
|  | 12 to <18 | | $ 375.18 | $ 358.88 | $ 392.17 |  |
| **Gender** | |  |  |  |  |  |
|  | Female |  | $ 362.06 | $ 334.99 | $ 366.65 | <0.001 |
|  | Male |  | $ 389.20 | $ 361.30 | $ 390.29 |  |
| **Co-NSSF** | |  |  |  |  |  |
|  | No |  | $ 371.82 | $ 358.63 | $ 385.48 | 0.03 |
|  | Yes |  | $ 353.97 | $ 337.14 | $ 371.63 |  |
| **Diagnosis code** | | |  |  |  |  |
|  | S00 | Superficial injury of head | $ 151.40 | $ 141.51 | $ 161.97 |  |
|  | S01 | Open wound of scalp | $ 172.79 | $ 161.31 | $ 185.12 |  |
|  | S02 | Fracture of skull and facial bones | $ 967.87 | $ 858.17 | $ 1,091.60 |  |
|  | S03 | Dislocation, sprain and strain of joints and ligaments of head | $ 169.85 | $ 123.84 | $ 232.94 |  |
|  | S05 | Injury of eye and orbit | $ 335.63 | $ 277.13 | $ 406.47 |  |
|  | S06 | Intracranial injury | $ 635.37 | $ 578.36 | $ 698.06 |  |
|  | S09 | Other and unspecified injuries of head | $ 386.22 | $ 329.90 | $ 452.14 |  |
|  | S10 | Superficial injury of neck | $ 153.96 | $ 111.49 | $ 212.64 |  |
|  | S20 | Superficial injury of thorax | $ 164.24 | $ 117.91 | $ 228.77 |  |
|  | S30 | Superficial injury of abdomen, lower back and pelvis | $ 127.18 | $ 109.46 | $ 147.75 |  |
|  | S40 | Superficial injury of shoulder and upper arm | $ 112.33 | $ 95.55 | $ 132.04 |  |
|  | S42 | Fracture of shoulder and upper arm | $ 1,583.51 | $ 1,429.39 | $ 1,754.08 |  |
|  | S43 | Dislocation, sprain and strain of joints and ligaments of shoulder girdle | $ 524.06 | $ 370.81 | $ 740.63 |  |
|  | S50 | Superficial injury of forearm | $ 127.97 | $ 114.73 | $ 142.75 |  |
|  | S51 | Open wound of forearm | $ 203.26 | $ 145.90 | $ 283.16 |  |
|  | S52 | Fracture of forearm | $ 1,077.61 | $ 1,013.94 | $ 1,145.39 |  |
|  | S53 | Dislocation, sprain and strain of joints and ligaments of elbow | $ 294.39 | $ 250.18 | $ 346.37 |  |
|  | S60 | Superficial injury of wrist and hand | $ 88.62 | $ 84.17 | $ 93.31 |  |
|  | S61 | Open wound of wrist and hand | $ 268.97 | $ 237.65 | $ 304.42 |  |
|  | S62 | Fracture at wrist and hand level | $ 551.97 | $ 506.29 | $ 601.78 |  |
|  | S63 | Dislocation, sprain and strain of joints and ligaments at wrist and hand level | $ 137.96 | $ 114.88 | $ 165.67 |  |
|  | S69 | Other and unspecified injuries of wrist and hand | $ 720.54 | $ 550.21 | $ 943.50 |  |
|  | S70 | Superficial injury of hip and thigh | $ 159.51 | $ 122.11 | $ 208.35 |  |
|  | S72 | Fracture of femur | $ 2,607.12 | $ 2,306.30 | $ 2,947.17 |  |
|  | S73 | Dislocation, sprain and strain of joint and ligaments of hip | $ 737.01 | $ 510.15 | $ 1,064.86 |  |
|  | S80 | Superficial injury of lower leg | $ 102.21 | $ 93.72 | $ 111.47 |  |
|  | S81 | Open wound of lower leg | $ 221.49 | $ 178.38 | $ 275.04 |  |
|  | S82 | Fracture of lower leg, including ankle | $ 1,406.84 | $ 1,276.78 | $ 1,550.14 |  |
|  | S83 | Dislocation, sprain and strain of joints and ligaments of knee | $ 1,920.23 | $ 1,547.51 | $ 2,382.96 |  |
|  | S90 | Superficial injury of ankle and foot | $ 93.99 | $ 87.90 | $ 100.49 |  |
|  | S91 | Open wound of ankle and foot | $ 188.67 | $ 149.01 | $ 238.89 |  |
|  | S92 | Fracture of foot, except ankle | $ 509.38 | $ 401.34 | $ 646.45 |  |
|  | S93 | Dislocation, sprain and strain of joints and ligaments at ankle and foot level | $ 153.38 | $ 125.09 | $ 188.07 |  |
|  | T00 | Superficial injuries involving multiple body regions | $ 731.43 | $ 593.00 | $ 902.17 |  |
|  | T07 | Unspecified multiple injuries | $ 1,293.23 | $ 1,017.39 | $ 1,643.86 |  |
|  | T14 | Injury of unspecified body region | $ 495.02 | $ 422.80 | $ 579.58 |  |
|  | T17 | Foreign body in respiratory tract | $ 772.24 | $ 616.16 | $ 967.87 |  |
|  | T18 | Foreign body in alimentary tract | $ 625.91 | $ 493.98 | $ 792.98 |  |
|  | T50 | Poisoning by, adverse effect of and underdosing of diuretics and other and unspecified drugs, medicaments and biological substances | $ 471.77 | $ 331.92 | $ 670.55 |  |
|  | T75 | Effects of other external causes | $ 546.65 | $ 382.80 | $ 780.71 |  |
|  | T78 | Adverse effects, not elsewhere classified | $ 507.40 | $ 364.34 | $ 706.70 |  |

**Table S4. Rank, Mean cost, and Total Cost of Child Injury in Lebanon – ED, Admit, and Total**

|  |  | **Emergency Costs by Diagnosis Code** | | | | | |  | **Admitted Costs by Diagnosis Code** | | | | | |  | **Overall Costs by Diagnosis Code** | | | | | |
| --- | --- | --- | --- | --- | --- | --- | --- | --- | --- | --- | --- | --- | --- | --- | --- | --- | --- | --- | --- | --- | --- |
|  |  | **Injury** | | **Mean Unit  Cost** | | **ED Total Cost** | |  | **Injury** | | **Mean Unit Cost** | | **Admit Total Cost** | |  | **Injury** | | **Mean Unit Cost** | | **Overall  Total Cost** | |
| **Diagnosis code category** | | **Rank** | **N** | **Rank** | **Unit $** | **Rank** | **Total $** |  | **Rank** | **N** | **Rank** | **Unit $** | **Rank** | **Total $** |  | **Rank** | **N** | **Rank** | **Unit $** | **Rank** | **Total $** |
| S52 | Fracture of forearm | 19 | 1,013 | 5 | $200 | 15 | $202,742 |  | 1 | 1041 | 9 | $1,593 | 1 | $1,658,490 |  | 12 | 2,054 | 9 | $1,593 | 1 | $1,861,232 |
| S01 | Open wound of scalp | 3 | 9,232 | 11 | $170 | 1 | $1,565,213 |  | 3 | 608 | 31 | $300 | 11 | $182,300 |  | 3 | 9,840 | 31 | $300 | 2 | $1,747,513 |
| S60 | Superficial injury of wrist and hand | 1 | 13,167 | 37 | $114 | 2 | $1,503,923 |  | 2 | 965 | 40 | $138 | 16 | $133,645 |  | 1 | 14,132 | 40 | $138 | 3 | $1,637,567 |
| S90 | Superficial injury of ankle and foot | 2 | 9,572 | 39 | $113 | 3 | $1,083,490 |  | 4 | 545 | 39 | $141 | 20 | $76,952 |  | 2 | 10,117 | 39 | $141 | 4 | $1,160,442 |
| S82 | Fracture of lower leg, including ankle | 31 | 327 | 3 | $221 | 27 | $72,263 |  | 8 | 383 | 4 | $2,759 | 2 | $1,056,584 |  | 24 | 710 | 4 | $2,759 | 5 | $1,128,845 |
| S00 | Superficial injury of head | 4 | 6,131 | 13 | $160 | 4 | $979,089 |  | 5 | 525 | 35 | $256 | 15 | $134,502 |  | 4 | 6,656 | 35 | $256 | 6 | $1,113,592 |
| S06 | Intracranial injury | 12 | 1,648 | 6 | $196 | 10 | $323,611 |  | 6 | 467 | 15 | $1,187 | 5 | $554,400 |  | 10 | 2,115 | 15 | $1,187 | 7 | $878,010 |
| S72 | Fracture of femur | 41 | 36 | 1 | $608 | 37 | $21,879 |  | 12 | 212 | 3 | $3,785 | 3 | $802,458 |  | 36 | 248 | 3 | $3,785 | 8 | $824,337 |
| S42 | Fracture of shoulder and upper arm | 32 | 297 | 2 | $233 | 29 | $69,146 |  | 9 | 329 | 5 | $2,136 | 4 | $702,906 |  | 25 | 626 | 5 | $2,136 | 9 | $772,051 |
| S62 | Fracture at wrist and hand level | 14 | 1,559 | 20 | $149 | 13 | $231,690 |  | 7 | 437 | 18 | $1,085 | 6 | $474,088 |  | 13 | 1,996 | 18 | $1,085 | 10 | $705,778 |
| S61 | Open wound of wrist and hand | 9 | 2,630 | 14 | $157 | 8 | $413,783 |  | 14 | 205 | 24 | $698 | 13 | $143,083 |  | 9 | 2,835 | 24 | $698 | 11 | $556,867 |
| S63 | Dislocation, sprain and strain of joints and ligaments at wrist and hand level | 5 | 4,426 | 35 | $116 | 5 | $513,441 |  | 19 | 105 | 30 | $346 | 31 | $36,305 |  | 6 | 4,531 | 30 | $346 | 12 | $549,745 |
| S80 | Superficial injury of lower leg | 6 | 4,327 | 34 | $117 | 6 | $508,259 |  | 10 | 289 | 41 | $127 | 30 | $36,794 |  | 5 | 4,616 | 41 | $127 | 13 | $545,053 |
| S83 | Dislocation, sprain and strain of joints and ligaments of knee | 15 | 1,291 | 23 | $136 | 18 | $175,792 |  | 20 | 92 | 2 | $3,912 | 8 | $359,879 |  | 16 | 1,383 | 2 | $3,912 | 14 | $535,670 |
| T07 | Unspecified multiple injuries | 37 | 152 | 8 | $176 | 35 | $26,825 |  | 24 | 74 | 1 | $6,201 | 7 | $458,840 |  | 38 | 226 | 1 | $6,201 | 15 | $485,665 |
| S93 | Dislocation, sprain and strain of joints and ligaments at ankle and foot level | 7 | 3,758 | 36 | $115 | 7 | $430,529 |  | 22 | 84 | 26 | $600 | 26 | $50,424 |  | 7 | 3,842 | 26 | $600 | 16 | $480,953 |
| S09 | Other and unspecified injuries of head | 10 | 1,921 | 15 | $157 | 11 | $302,119 |  | 16 | 148 | 20 | $934 | 14 | $138,289 |  | 11 | 2,069 | 20 | $934 | 17 | $440,407 |
| S50 | Superficial injury of forearm | 8 | 2,953 | 33 | $122 | 9 | $359,301 |  | 13 | 210 | 34 | $271 | 25 | $56,963 |  | 8 | 3,163 | 34 | $271 | 18 | $416,264 |
| S02 | Fracture of skull and facial bones | 33 | 292 | 9 | $172 | 32 | $50,283 |  | 11 | 221 | 10 | $1,591 | 9 | $351,547 |  | 31 | 513 | 10 | $1,591 | 19 | $401,830 |
| T14 | Injury of unspecified body region | 20 | 959 | 7 | $184 | 17 | $176,848 |  | 15 | 166 | 17 | $1,097 | 12 | $182,051 |  | 19 | 1,125 | 17 | $1,097 | 20 | $358,898 |
| T00 | Superficial injuries involving multiple body regions | 28 | 458 | 4 | $206 | 24 | $94,559 |  | 21 | 90 | 6 | $2,108 | 10 | $189,678 |  | 28 | 548 | 6 | $2,108 | 21 | $284,236 |
| S30 | Superficial injury of abdomen, lower back and pelvis | 11 | 1,699 | 24 | $136 | 14 | $231,058 |  | 18 | 108 | 36 | $237 | 34 | $25,620 |  | 14 | 1,807 | 36 | $237 | 22 | $256,678 |
| S40 | Superficial injury of shoulder and upper arm | 13 | 1,599 | 21 | $145 | 12 | $231,696 |  | 22 | 84 | 37 | $166 | 37 | $13,966 |  | 15 | 1,683 | 37 | $166 | 23 | $245,662 |
| S53 | Dislocation, sprain and strain of joints and ligaments of elbow | 17 | 1,114 | 28 | $127 | 19 | $141,067 |  | 17 | 109 | 23 | $725 | 19 | $78,990 |  | 17 | 1,223 | 23 | $725 | 24 | $220,056 |
| S91 | Open wound of ankle and foot | 16 | 1,151 | 17 | $156 | 16 | $179,185 |  | 29 | 61 | 29 | $509 | 32 | $31,059 |  | 18 | 1,212 | 29 | $509 | 25 | $210,244 |
| S05 | Injury of eye and orbit | 21 | 822 | 18 | $155 | 20 | $127,012 |  | 26 | 69 | 14 | $1,193 | 18 | $82,316 |  | 21 | 891 | 14 | $1,193 | 26 | $209,327 |
| S69 | Other and unspecified injuries of wrist and hand | 18 | 1,037 | 38 | $114 | 22 | $118,104 |  | 31 | 57 | 12 | $1,251 | 21 | $71,335 |  | 20 | 1,094 | 12 | $1,251 | 27 | $189,439 |
| S81 | Open wound of lower leg | 22 | 725 | 10 | $170 | 21 | $122,971 |  | 27 | 68 | 25 | $614 | 27 | $41,752 |  | 22 | 793 | 25 | $614 | 28 | $164,723 |
| S92 | Fracture of foot, except ankle | 29 | 414 | 29 | $127 | 31 | $52,395 |  | 28 | 67 | 11 | $1,375 | 17 | $92,125 |  | 32 | 481 | 11 | $1,375 | 29 | $144,520 |
| S43 | Dislocation, sprain and strain of joints and ligaments of shoulder girdle | 26 | 494 | 19 | $151 | 26 | $74,659 |  | 34 | 34 | 7 | $2,034 | 22 | $69,153 |  | 30 | 528 | 7 | $2,034 | 30 | $143,811 |
| T17 | Foreign body in respiratory tract | 27 | 469 | 32 | $123 | 30 | $57,844 |  | 25 | 70 | 19 | $978 | 23 | $68,443 |  | 29 | 539 | 19 | $978 | 31 | $126,287 |
| S03 | Dislocation, sprain and strain of joints and ligaments of head | 23 | 716 | 16 | $156 | 23 | $111,529 |  | 32 | 43 | 32 | $289 | 39 | $12,437 |  | 23 | 759 | 32 | $289 | 32 | $123,966 |
| S70 | Superficial injury of hip and thigh | 25 | 565 | 27 | $127 | 28 | $71,564 |  | 34 | 34 | 13 | $1,220 | 28 | $41,495 |  | 27 | 599 | 13 | $1,220 | 33 | $113,059 |
| T18 | Foreign body in alimentary tract | 30 | 341 | 31 | $124 | 33 | $42,139 |  | 29 | 61 | 16 | $1,119 | 24 | $68,253 |  | 33 | 402 | 16 | $1,119 | 34 | $110,392 |
| S20 | Superficial injury of thorax | 24 | 570 | 25 | $132 | 25 | $75,438 |  | 36 | 30 | 33 | $278 | 40 | $8,329 |  | 26 | 600 | 33 | $278 | 35 | $83,768 |
| S73 | Dislocation, sprain and strain of joint and ligaments of hip | 34 | 263 | 40 | $95 | 36 | $25,033 |  | 40 | 23 | 8 | $1,734 | 29 | $39,889 |  | 34 | 286 | 8 | $1,734 | 36 | $64,922 |
| S51 | Open wound of forearm | 36 | 216 | 12 | $167 | 34 | $36,167 |  | 39 | 26 | 27 | $525 | 38 | $13,659 |  | 37 | 242 | 27 | $525 | 37 | $49,826 |
| T78 | Adverse effects, not elsewhere classified | 35 | 239 | 41 | $91 | 38 | $21,797 |  | 33 | 35 | 22 | $768 | 33 | $26,868 |  | 35 | 274 | 22 | $768 | 38 | $48,664 |
| T75 | Effects of other external causes | 40 | 51 | 26 | $128 | 41 | $6,512 |  | 37 | 28 | 21 | $791 | 35 | $22,162 |  | 41 | 79 | 21 | $791 | 39 | $28,674 |
| T50 | Poisoning by, adverse effect of and underdosing of diuretics and other and unspecified drugs, medicaments and biological substances | 39 | 75 | 30 | $126 | 40 | $9,478 |  | 37 | 28 | 28 | $514 | 36 | $14,398 |  | 40 | 103 | 28 | $514 | 40 | $23,876 |
| S10 | Superficial injury of neck | 38 | 145 | 22 | $144 | 39 | $20,943 |  | 41 | 17 | 38 | $162 | 41 | $2,758 |  | 39 | 162 | 38 | $162 | 41 | $23,701 |
|  |  |  |  |  |  |  |  |  |  |  |  |  |  |  |  |  |  |  |  |  |  |
