# Supplementary material for: Incidence and direct medical costs of child injuries in Lebanon (2012–2016): Evidence from closed insurance claims analysis
Source: PLoS One. 2026 Jul 21;21(7):e0353679. doi: 10.1371/journal.pone.0353679 (PMC13387535; doi:10.1371/journal.pone.0353679)
Supplement: Attachment — Submitted filename: RTR_Plosone_Final.pdf [file pone.0353679.s005.pdf]

**29 May 2026**

Dear Editors and Reviewers,

On behalf of my co-authors, thank you for the careful and constructive reviews of our manuscript, **“Incidence and direct medical costs of child injuries in Lebanon (2012–2016): Evidence from closed insurance claims analysis.”** We are grateful for the opportunity to revise our submission.

In this revision, we have implemented changes to improve clarity, transparency, and readability. Specifically, we clarified our definition of pediatric patients (<18 years), incorporated age-group categorization in the descriptive presentation, revised statistical significance wording, and reformatted and updated tables (including revised copies of Tables 2 and 3). We also strengthened the Discussion and Conclusion and clarified limitations inherent to closed insurance-claims data, including constrained clinical detail and the inability to measure indirect costs or injury mechanisms.

We provide below a point-by-point response to each comment and indicate where changes were made in the revised manuscript and tables. We appreciate the reviewers’ guidance in improving the manuscript and hope the revised version meets the journal’s standards.

Sincerely,
**Dr. Samar Al-Hajj**
Corresponding Author
American University of Beirut
Email: sh137@aub.edu.lb

Incidence and direct medical costs of child injuries in Lebanon (2012-2016): Evidence from closed insurance claims analysis

When submitting your revision, we need you to address these additional requirements.
1. Please ensure that your manuscript meets PLOS ONE's style requirements, including those for file naming. The PLOS ONE style templates can be found at https://journals.plos.org/plosone/s/file?id=wjVg/PLOSOne_formatting_sample_main_body.pdf and https://journals.plos.org/plosone/s/file?id=ba62/PLOSOne_formatting_sample_title_authors_affiliations.pdf

Thank you. We have revised the manuscript, tables and figures to be in the journals requirements.

2. We note that you have indicated that there are restrictions to data sharing for this study. For studies involving human research participant data or other sensitive data, we encourage authors to share de-identified or anonymized data. However, when data cannot be publicly shared for ethical reasons, we allow authors to make their data sets available upon request. For information on unacceptable data access restrictions, please see http://journals.plos.org/plosone/s/data-availability#loc-unacceptable-data-access-restrictions.

Before we proceed with your manuscript, please address the following prompts:

a) If there are ethical or legal restrictions on sharing a de-identified data set, please explain them in detail (e.g., data contain potentially identifying or sensitive patient information, data are owned by a third-party organization, etc.) and who has imposed them (e.g., a Research Ethics Committee or Institutional Review Board, etc.). Please also provide contact information for a data access committee, ethics committee, or other institutional body to which data requests may be sent.

b) If there are no restrictions, please upload the minimal anonymized data set necessary to replicate your study findings to a stable, public repository and provide us with the relevant URLs, DOIs, or accession numbers. Please see http://www.bmj.com/content/340/bmj.c181.long for guidelines on how to de-identify and prepare clinical data for publication. For a list of recommended repositories, please see https://journals.plos.org/plosone/s/recommended-repositories. You also have the option of uploading the data as Supporting Information files, but we would recommend depositing data directly to a data repository if possible.

Please update your Data Availability statement in the submission form accordingly.

*Thank you for your comment. Data will be made available upon reasonable request from the corresponding author (sh137@aub.edu.lb). De-identified data cannot be made publicly*

*available as they are acquired from four Third-Party Administrators for health insurance companies, and thus are not owned by the authors' of the study.*

3. Please include a new copy of Tables 2 and 3 in your manuscript; the current table is difficult to read. Please follow the link for more information: https://journals.plos.org/plosone/s/tables

*Thank you for your comment. We have modified Tables 2 and 3 to meet the requirements.*

4. If the reviewer comments include a recommendation to cite specific previously published works, please review and evaluate these publications to determine whether they are relevant and should be cited. There is no requirement to cite these works unless the editor has indicated otherwise.

*Thank you for your comment. Well noted.*

Reviewer #1: Abstract
1). A clear definition of a paediatric patient should be articulated, as a paediatric patient is an individual who is a minor, typically defined as being under 15 years of age (and in some jurisdictions, under 18 or 21 years of age).

*Thank you for your comment. Please note that within the context of this paper, Paediatric patients are defined as children below the age of 18 (stated In the Method section).*

2). The results would benefit from categorisation into specific age groups:

- neonates (birth to 28 days),
- infants (29 days to under 2 years),
- children (2 to under 12 years), and
- adolescents (12 to 18 or 21 years, depending on the authors' definition).

*The breakdown of the population represented has been categorized by age group in Table 1. Due to sufficient data in the administrative dataset neonates and infants were combined into one category, children 2 to under 12, and adolescents 12 to under 18 are now listed. Further, we have created a new figure 2 to demonstrate the contributions of injuries to each of these age groups stratified by disposition (ED vs Admitted) to the costs in our results.*

3). Cost presentation would also be enhanced by categorizing the data, rather than currently providing a total for all claims, while specific types of injuries are presented as proportions.

Incidence and direct medical costs of child injuries in Lebanon (2012-2016): Evidence from closed insurance claims analysis

*Thank you for this suggestion. Cost presentation is categorized in Table 3. This is limited by the nature of the data from the closed insurance claims where the majority of the claims were not categorized and simply billed as a global charge. Particularly for the 86% of cases that were seen and discharged after emergency department treatment*

4). The conclusion requires revision; for instance, the results do not provide any information regarding interventions related to pediatric safety and security or indirect costs.
*Thank you for your comment. The abstract has been revised to align with your suggestions.*

Introduction
5). A clear definition of a pediatric patient should be articulated.
*Thank you for your comment. Please note that within the context of this paper, Paediatric patients are defined as children below the age of 18 (stated In the Method section).*

6). Authors have cited many articles; for example, on page 3, last paragraph [9, 10, 11, 12, 13, ...], there is a lack of summarized information on what the articles explored and their link to pediatric injury in EMR.

*Thank you for your comment. We have retained the most relevant literature that supports the introduction. Some statements are supported by multiple references because there are relatively few studies addressing this topic, and these references collectively provide the necessary context and evidence for the points mentioned.*

7). Content in some of the paragraphs keep on repeating, from page 3 last paragraph, page 4 first paragraph and the last paragraph.

*Thank you for your comment. We have revised the introduction to remove any repetition.*

8). The introduction misses out on the country context regarding existing policies, as well as the safety and security of children aged below 18 years.

*Thank you for this comment. We have added contextual information on Lebanon's policies and child safety environment, noting the lack of a comprehensive national child injury prevention strategy and the limited enforcement of safety regulations.*

9). The context of health financing is not clearly articulated, encompassing aspects such as user fees, health insurance, and funding from development partners. Does health insurance cover all the medical costs or patients have to pay some co-payments, for health commodities and admission?

Incidence and direct medical costs of child injuries in Lebanon (2012-2016): Evidence from closed insurance claims analysis

*Thank you for this comment. Lebanon has a mixed health financing system that includes public coverage, private health insurance, and out-of-pocket payments, with private insurance commonly involving co-payments for services such as emergency care and hospital admission. This study focuses on direct medical costs derived from private insurance closed claims and therefore reflects insurer-reimbursed costs only. Costs borne directly by patients or covered through other financing mechanisms were not captured and represent a limitation of this analysis. This was mentioned in the discussion, but additional information has been added to provide context in the introduction.*

10). Furthermore, the service delivery context requires clarification. Given that some of the results have been presented in relation to various departments, it is necessary to explicitly delineate the service delivery mechanisms.

*Thank you for this comment. In Lebanon, pediatric injury care is primarily delivered through hospital-based emergency departments, with more severe cases requiring inpatient admission and specialized services. We have clarified the service delivery context in the introduction to explain how injuries are managed across different departments. The line item costs, when provided, were categorized by two clinicians with trauma care experience. As stated on page 6 - Each charge was grouped into a service category based on the approved procedure or service by trained clinicians (HM, EP).*

Methods

11). First paragraph time frame shown is not clear ...... " De-identified, injury- related, closed insurance claims were obtained from four leading Third Party Administrative (TPA) companies for children (<18 years) with International Classification of Diseases (ICD-10) codes corresponding to Injury, poisoning and certain other consequences of external causes (S00-T88) between the period of 01 January 2012 through 31 December 2016 on September 15, 2018...."

*Thank for your pointing out this clerical error. It has been corrected*

Ethics declaration

12). The tern "Ethics declatarion" needs to be corrected.

*Thank you for your comment. This section has been revised.*

13). Revise the ethics to align with journal requirement

*Thank you for your comment. This section has been revised.*

Methods

14). The section lacks information on

i). Currency conversion USD/LBP (Report the dates of the estimated resource quantities and unit costs, plus the currency and year of conversion).

Incidence and direct medical costs of child injuries in Lebanon (2012-2016): Evidence from closed insurance claims analysis

All costs in this dataset were reported in US dollars as is common in Lebanon. No currency conversion was done. The costs were also analyzed as reported over the five years of the study without adjustment for inflation. Since this is analysis of historical data from closed insurance claims we could not estimate the precise date of processing each claim, only the date of the service provided. Furthermore, in the period of study the inflation rate in Lebanon was low experiencing deceleration and finally deflation with the Consumer Price Index varied from a high of 6.58 in 2012 declining each year to a deflationary period in (2015,-3.75%) and (2016, -0.78%). The cumulative average inflation was only 1.82%. There was a fixed conversion rate of 1USD = LBP 1500 during the period of interest.

ii). Clear description of characteristics of the study population (such as age range, demographics, or clinical characteristics)
The study population is outlined in Table 1 and in the methods section

iii). Setting and location - Provide relevant contextual information that may influence findings.
*This is a secondary analysis of an insurance dataset. There is no contextual information regarding the settings and location other than all data is reported from hospitals. This has been further clarified in the introduction/*

iv). State the perspective(s) adopted by the study and why chosen
Thank you for this comment. We have revised the Methods to explicitly state the study perspective. This analysis adopts a **healthcare payer/claims (third-party administrator/insurer) perspective**, estimating **direct medical costs captured in closed insurance claims** (i.e., allowed/approved amounts recorded in the claims database). We chose this perspective because our data source is administrative claims from multiple TPAs, which reliably capture reimbursed direct medical expenditures, and because payer-perspective estimates are directly relevant for budgeting, reimbursement policy, and identifying high-cost injury categories for prevention and quality improvement. We also clarify that **indirect costs** (e.g., caregiver productivity loss, transportation, out-of-pocket expenditures not captured in claims) are **not included**, and therefore the estimates should not be interpreted as societal costs.

v). Describe how costs were valued, adjusted etc.
*Costs were taken as reported in US Dollars. They are not adjusted for inflation. As indicated above the period of study chosen was that of high price stability (average inflation of 1.8% over 5 years)*

vi). Describe any methods for analysing or statistically transforming data, any extrapolation methods etc.
*Additional information was added to the methods section to state that* given only positive cost and positive skew of the data, we used a log-transformation to appropriately model the data with regression analysis

Incidence and direct medical costs of child injuries in Lebanon (2012-2016): Evidence from closed insurance claims analysis

vii). The insurance claim contains various levels of service delivery, which vary in terms of charges/reimbursements; aggregation of these levels could be misleading.
The majority (76%) of charges were not categorized and simply presented as a global charge. All charges were for hospital-based emergency department or inpatient care where most injury care is provided in Lebanon

viii). Describe any methods used for estimating how the results of the study vary for subgroups.
*We have added subgroup analysis by age groups (less than 2 years, 2 to less than 12, 12 to less than 18)*

ix). Clear stating the cost comparison being presented in the results section (male vs female, facility level [such as Primary Healthcare, Advanced Specialized Care, Super Specialized Care, etc], health facility departments etc)
The only comparison being made in the results section was between male and female presentations and between Emergency Department treatment vs inpatient hospital treatment costs. All charges were for hospital-based emergency department or inpatient care where most injury care is provided in Lebanon. No data was presented from primary health centers or other non-hospital facilities

15). Data analysis
i). How were patterns assessed and presented in the results section? Also pediatric visits needs clarity in the methods section, for the 2nd or 3rd visits were these linked to the single case of injury or multiple cases of injury?

As stated in the Data Analysis section we only use the first visit per patient for the regression analysis. We also summarize each injury (diagnosis code) at separate visits descriptively

ii). Explain the reason behind decision to present the findings by gender and not by any other categorization (age, health facility level etc)

*The convention of presenting the data by sex was maintained. We have added the breakdown of age groups for pediatric injury presentations. All data is from hospital visits only.*

iii). A log-transformed generalized liner model was used to analyze costs of adult injury, does this mean claim data was filtered to reflect 12 to 18 or 21 years or all the pediatric patient?

Thank you for pointing out this typo. We have updated to read “cost of pediatric injury” since this data focuses on children, < 18 yr.

Incidence and direct medical costs of child injuries in Lebanon (2012-2016): Evidence from closed insurance claims analysis

iV) A p-value of 0.05 was considered statistically significant. This should be amended to a p-value of less than 0.05 was considered statistically significant.

Thank you. We have changed the wording

v). Use of mean and median: was the claim data normally distributed?
Given only positive cost and positive skew of the data, we used a log-transformation to appropriately model the data with regression analysis.

Results
16). Amend Table 1: where proportion of the Co-NSSF is presented and merge the cells Yes/Yes or No/No

*Thank you for your comment, this has been fixed.*

17). Table 1: A presentation of the top 5 injuries and least 5 can be done and the rest of type of injuries can be presented as supplementary tables
*Thank you for this suggestion. We have split Table 1 into two tables – new Table 1 – characteristics of children presenting with injuries, new Table 2 – diagnosis codes of injury presentations stratified by sex*

18). Presentation of the costs can also be in terms of service delivery type
We agree and have presented costs by service delivery type in **Table 3** (e.g., ED visit charges and ancillary services). We note an important data limitation: in the claims dataset, **more than three-quarters of ED-based costs are recorded as an aggregated (lump-sum) ED charge without itemized service breakdown**, which constrains finer disaggregation of ED costs by specific service components. We have clarified this limitation in the manuscript and reflected it in our interpretation of the service-type cost patterns.

19). The table should be properly formatted for easy follow-up.
*The tables have been reformatted to more easily display*

20). Table 2. Child Injury incidence, unit cost, and overall. Use of child, pediatric patient needs to be consistent as the analysis did not categorize pediatric into age groups [(birth to 28 days), infants (29 days to under 2 years), children (2 to under 12 years), and adolescents (12 to 18 or 21 years]
*We have revised the text to be more consistent in the use of child injury and have provided the breakdown of ages in Table 1, Supplemental Tables 1-3, and Figure 2*

21). Supplementary Tables should be clearly numbered and referenced.

*Thank you for your comment. We fixed the referencing.*

Incidence and direct medical costs of child injuries in Lebanon (2012-2016): Evidence from closed insurance claims analysis

22). Use of mean and median: for example page 24 last sentence .....
"Inpatient injury costs varied by age as well as diagnosis with median costs for orthopedic treatments for extremity fractures or dislocations being 3x – 7x cost of other injuries treated (S2 Table)"..... The methods section did not clarify a comparison will be made between orthopedic care to other injuries care provided in Lebanon.
Thank you for noting this. We have **corrected the text** to clarify that the statement was referring to the **geometric mean (model-based estimate)** rather than the median. We also clarify that **we did not prespecify a formal analysis comparing "orthopedic care" to "other injury care"** as a distinct analytic aim. Instead, the statement reflects an **empirical finding** observed in both (i) the **descriptive cost distributions** (Figure 2) and (ii) the **multivariable regression results**, where **orthopedic injury diagnoses (including extremity fractures and dislocations)** were associated with substantially higher inpatient costs relative to other injury diagnoses. We have revised the wording in the Results/Discussion to avoid implying a pre-planned orthopedic-versus-other comparison and to ensure consistent interpretation of model outputs.

Discussion and conclusion
23). Discussion and conclusion should be strengthened
*Thank you for your comment. We have revised the discussion section and expanded the discussion of generalizability and limitations, we have elaborated on the limitations of the claims-based dataset.*

24). Conclusion should be revised

*Thank you for your comment. We have revised the conclusion.*

Acknowledgement
25). Be revised as, data are own by an institution, authors might have received financial resources to undertake the work (not directly to fund the study), technical support etc
There were no financial resources provided to undertake this work. Technical support was provided by academic colleagues who are named co-authors

Incidence and direct medical costs of child injuries in Lebanon (2012-2016): Evidence from closed insurance claims analysis

Reviewer #2: The data is nearly ten years old on a subject of trauma. Trauma related findings and information shared have no new information. In fact cost is mixed up with injuries in a unimpressive style. Data is taken from insurance companies thus even injury pattern and its impact on the children and families can not be determined.

We thank you for this comment. We respectfully disagree that the work lacks novelty. There is **very limited published evidence on the direct medical costs of pediatric injuries from the Eastern Mediterranean Region**, and to our knowledge there are no Lebanon-wide estimates derived from multi-payer closed claims. By leveraging data from multiple third-party administrators covering a substantial share of the insured market, our analysis provides a **rare national-scale benchmark** of pediatric injury incidence patterns and direct medical costs, which can inform priority-setting and future evaluations of cost-effective injury prevention and care.

We acknowledge that the study period (2012–2016) is not recent. This reflects (i) the substantial time required to obtain approvals from multiple independent insurance stakeholders and (ii) stakeholders' preference to share **historical closed claims** rather than more recent claims that could still be subject to appeal or other administrative processes. Importantly, the selected period also corresponds to **exceptionally low inflation and relative price stability in Lebanon**, improving internal comparability of cost estimates across years and offering a valuable **pre-crisis baseline** against which post-2019 patterns may be interpreted in future work, given the hyperinflationary context after 2019.

There is unnecessary verbosity in the introduction. References are irrationally used. There are eight references in the discussion section put together in an unintelligent style like 30, 31,32,33 and 35, 36, 37, 38, 39, 40. It appears that authors are not keen interest in comparing with the reported literature. There are huge limitations already identified by the authors too.

Thank you for this comment. We agree that the original Introduction and Discussion required tightening and clearer engagement with the literature. In the revised manuscript, we have **substantially edited the Introduction to reduce verbosity**, focusing it on: (i) the burden of pediatric injuries, (ii) why estimating direct medical costs is important for policy and prevention, (iii) the evidence gap in Lebanon/EMR, and (iv) the specific contribution of multi-payer closed claims data.

We also **curated and reorganized the references** to improve the logic and readability of the Discussion. Where multiple studies were previously cited in long strings, we now cite **only the most directly relevant sources** and link each citation to a specific comparative point (e.g., incidence patterns, cost drivers, ED vs inpatient costs, and methodological differences across settings). In addition, we **expanded the comparison to published literature**, prioritizing evidence from the **Eastern Mediterranean Region and comparable LMIC contexts**, and we clarified how differences in health system structure, payer mix, and claims/billing practices may explain divergences across studies.

Finally, we retained the limitations section but revised it to ensure limitations are **clearly framed and proportionate** to the study’s contribution, and to avoid overstating generalizability beyond insured claims data.
