# Supplementary material for: Incidence and direct medical costs of child injuries in Lebanon (2012–2016): Evidence from closed insurance claims analysis
Source: PLoS One. 2026 Jul 21;21(7):e0353679. doi: 10.1371/journal.pone.0353679 (PMC13387535; doi:10.1371/journal.pone.0353679)
Supplement: Peer review history

# Peer review: Decision Letter 0

**DOI:** 10.1371/journal.pone.0353679.r001

**Authors:** Mabel Aoun

12 Dec 2025

PONE-D-25-26319

Incidence and direct medical costs of child injuries in Lebanon (2012-2016): Evidence from closed insurance claims analysis

PLOS One

Dear Dr. Al-Hajj,

Thank you for submitting your manuscript to PLOS ONE. After careful consideration, we feel that it has merit but does not fully meet PLOS ONE’s publication criteria as it currently stands. Therefore, we invite you to submit a revised version of the manuscript that addresses the points raised during the review process.

Two reviewers and the academic editor assessed the manuscript. The research question is very relevant and addressed with the appropriate methodology. However the data collection timeline extends back ten years as noted by one of the reviewers. The authors are requested to clarify this issue and provide a rationale supporting the inclusion of older data. They are also asked to provide a point-by-point response to reviewers.

Please submit your revised manuscript by Jan 26 2026 11:59PM. If you will need more time than this to complete your revisions, please reply to this message or contact the journal office at plosone@plos.org. When you're ready to submit your revision, log on to https://www.editorialmanager.com/pone/ and select the 'Submissions Needing Revision' folder to locate your manuscript file.

Please include the following items when submitting your revised manuscript:

- A rebuttal letter that responds to each point raised by the academic editor and reviewer(s). You should upload this letter as a separate file labeled 'Response to Reviewers'.
- A marked-up copy of your manuscript that highlights changes made to the original version. You should upload this as a separate file labeled 'Revised Manuscript with Track Changes'.
- An unmarked version of your revised paper without tracked changes. You should upload this as a separate file labeled 'Manuscript'.

If you would like to make changes to your financial disclosure, please include your updated statement in your cover letter. Guidelines for resubmitting your figure files are available below the reviewer comments at the end of this letter.

If applicable, we recommend that you deposit your laboratory protocols in protocols.io to enhance the reproducibility of your results. Protocols.io assigns your protocol its own identifier (DOI) so that it can be cited independently in the future. For instructions see: https://journals.plos.org/plosone/s/submission-guidelines#loc-laboratory-protocols. Additionally, PLOS ONE offers an option for publishing peer-reviewed Lab Protocol articles, which describe protocols hosted on protocols.io. Read more information on sharing protocols at https://plos.org/protocols?utm_medium=editorial-email&utm_source=authorletters&utm_campaign=protocols.

We look forward to receiving your revised manuscript.

Kind regards,

Mabel Aoun, MD, MPH

Academic Editor

PLOS One

Journal Requirements:

When submitting your revision, we need you to address these additional requirements.

1. Please ensure that your manuscript meets PLOS ONE's style requirements, including those for file naming. The PLOS ONE style templates can be found at

https://journals.plos.org/plosone/s/file?id=wjVg/PLOSOne_formatting_sample_main_body.pdf and

https://journals.plos.org/plosone/s/file?id=ba62/PLOSOne_formatting_sample_title_authors_affiliations.pdf

2. We note that you have indicated that there are restrictions to data sharing for this study. For studies involving human research participant data or other sensitive data, we encourage authors to share de-identified or anonymized data. However, when data cannot be publicly shared for ethical reasons, we allow authors to make their data sets available upon request. For information on unacceptable data access restrictions, please see http://journals.plos.org/plosone/s/data-availability#loc-unacceptable-data-access-restrictions.

Before we proceed with your manuscript, please address the following prompts:

a) If there are ethical or legal restrictions on sharing a de-identified data set, please explain them in detail (e.g., data contain potentially identifying or sensitive patient information, data are owned by a third-party organization, etc.) and who has imposed them (e.g., a Research Ethics Committee or Institutional Review Board, etc.). Please also provide contact information for a data access committee, ethics committee, or other institutional body to which data requests may be sent.

b) If there are no restrictions, please upload the minimal anonymized data set necessary to replicate your study findings to a stable, public repository and provide us with the relevant URLs, DOIs, or accession numbers. Please see http://www.bmj.com/content/340/bmj.c181.long for guidelines on how to de-identify and prepare clinical data for publication. For a list of recommended repositories, please see https://journals.plos.org/plosone/s/recommended-repositories. You also have the option of uploading the data as Supporting Information files, but we would recommend depositing data directly to a data repository if possible.

Please update your Data Availability statement in the submission form accordingly.

3. Please include a new copy of Tables 2 and 3 in your manuscript; the current table is difficult to read. Please follow the link for more information: https://journals.plos.org/plosone/s/tables

4. If the reviewer comments include a recommendation to cite specific previously published works, please review and evaluate these publications to determine whether they are relevant and should be cited. There is no requirement to cite these works unless the editor has indicated otherwise.

[Note: HTML markup is below. Please do not edit.]

Reviewers' comments:

Reviewer's Responses to Questions

**Comments to the Author**

1. Is the manuscript technically sound, and do the data support the conclusions?

The manuscript must describe a technically sound piece of scientific research with data that supports the conclusions. Experiments must have been conducted rigorously, with appropriate controls, replication, and sample sizes. The conclusions must be drawn appropriately based on the data presented.

Reviewer #1: Yes

Reviewer #2: Partly

**********

2. Has the statistical analysis been performed appropriately and rigorously?

Reviewer #1: Yes

Reviewer #2: No

**********

3. Have the authors made all data underlying the findings in their manuscript fully available?

The PLOS Data policy requires authors to make all data underlying the findings described in their manuscript fully available without restriction, with rare exception (please refer to the Data Availability Statement in the manuscript PDF file). The data should be provided as part of the manuscript or its supporting information, or deposited to a public repository. For example, in addition to summary statistics, the data points behind means, medians and variance measures should be available. If there are restrictions on publicly sharing data—e.g. participant privacy or use of data from a third party—those must be specified.

Reviewer #1: No

Reviewer #2: Yes

**********

4. Is the manuscript presented in an intelligible fashion and written in standard English?

PLOS ONE does not copyedit accepted manuscripts, so the language in submitted articles must be clear, correct, and unambiguous. Any typographical or grammatical errors should be corrected at revision, so please note any specific errors here.

Reviewer #1: Yes

Reviewer #2: No

**********

5. Review Comments to the Author

Please use the space provided to explain your answers to the questions above. You may also include additional comments for the author, including concerns about dual publication, research ethics, or publication ethics. (Please upload your review as an attachment if it exceeds 20,000 characters)

Reviewer #1: Abstract

1). A clear definition of a paediatric patient should be articulated, as a paediatric patient is an individual who is a minor, typically defined as being under 15 years of age (and in some jurisdictions, under 18 or 21 years of age).

2). The results would benefit from categorisation into specific age groups: neonates (birth to 28 days), infants (29 days to under 2 years), children (2 to under 12 years), and adolescents (12 to 18 or 21 years, depending on the authors' definition).

3). Cost presentation would also be enhanced by categorizing the data, rather than currently providing a total for all claims, while specific types of injuries are presented as proportions.

4). The conclusion requires revision; for instance, the results do not provide any information regarding interventions related to pediatric safety and security or indirect costs.

Introduction

5). A clear definition of a pediatric patient should be articulated.

6). Authors have cited many articles; for example, on page 3, last paragraph [9, 10, 11, 12, 13, ...], there is a lack of summarized information on what the articles explored and their link to pediatric injury in EMR.

7). Content in some of the paragraphs keep on repeating, from page 3 last paragraph, page 4 first paragraph and the last paragraph.

8). The introduction misses out on the country context regarding existing policies, as well as the safety and security of children aged below 18 years.

9). The context of health financing is not clearly articulated, encompassing aspects such as user fees, health insurance, and funding from development partners. Does health insurance cover all the medical costs or patients have to pay some co-payments, for health commodities and admission?

10). Furthermore, the service delivery context requires clarification. Given that some of the results have been presented in relation to various departments, it is necessary to explicitly delineate the service delivery mechanisms.

Methods

11). First paragraph time frame shown is not clear ...... " De-identified, injury- related, closed insurance claims were obtained from four leading Third Party Administrative (TPA) companies for children (<18 years) with International Classification of Diseases (ICD-10) codes corresponding to Injury, poisoning and certain other consequences of external causes (S00-T88) between the period of 01 January 2012 through 31 December 2016 on September 15, 2018...."

Ethics declaration

12). The tern "Ethics declatarion" needs to be corrected.

13). Revise the ethics to align with journal requirement

Methods

14). The section lacks information on

i). Currency conversion USD/LBP (Report the dates of the estimated resource quantities and unit costs, plus the currency and year of conversion).

ii). Clear description of characteristics of the study population (such as age range, demographics, or clinical characteristics)

iii). Setting and location - Provide relevant contextual information that may influence findings.

iv). State the perspective(s) adopted by the study and why chosen

v). Describe how costs were valued, adjusted etc.

vi). Describe any methods for analysing or statistically transforming data, any extrapolation methods etc.

vii). The insurance claim contains various levels of service delivery, which vary in terms of charges/reimbursements; aggregation of these levels could be misleading.

viii). Describe any methods used for estimating how the results of the study vary for subgroups.

ix). Clear stating the cost comparison being presented in the results section (male vs female, facility level [such as Primary Healthcare, Advanced Specialized Care, Super Specialized Care, etc], health facility departments etc)

15). Data analysis

i). How were patterns assessed and presented in the results section? Also pediatric visits needs clarity in the methods section, for the 2nd or 3rd visits were these linked to the single case of injury or multiple cases of injury?

ii). Explain the reason behind decision to present the findings by gender and not by any other categorization (age, health facility level etc)

iii). A log-transformed generalized liner model was used to analyze costs of adult injury, does this mean claim data was filtered to reflect 12 to 18 or 21 years or all the pediatric patient?

iV) A p-value of 0.05 was considered statistically significant. This should be amended to a p-value of less than 0.05 was considered statistically significant.

v). Use of mean and median: was the claim data normally distributed?

Results

16). Amend Table 1: where proportion of the Co-NSSF is presented and merge the cells Yes/Yes or No/No

17). Table 1: A presentation of the top 5 injuries and least 5 can be done and the rest of type of injuries can be presented as supplementary tables

18). Presentation of the costs can also be in terms of service delivery type

19). The table should be properly formatted for easy follow-up.

20). Table 2. Child Injury incidence, unit cost, and overall. Use of child, pediatric patient needs to be consistent as the analysis did not categorize pediatric into age groups [(birth to 28 days), infants (29 days to under 2 years), children (2 to under 12 years), and adolescents (12 to 18 or 21 years]

21). Supplementary Tables should be clearly numbered and referenced

22). Use of mean and median: for example page 24 last sentence ..... "Inpatient injury costs varied by age as well as diagnosis with median costs for orthopedic treatments for extremity fractures or dislocations being 3x – 7x cost of other injuries treated (S2 Table)"..... The methods section did not clarify a comparison will be made between orthopedic care to other injuries care provided in Lebanon.

Discussion and conclusion

23). Discussion and conclusion should be strengthened

24). Conclusion should be revised

Acknowledgement

25). Be revised as, data are own by an institution, authors might have received financial resources to undertake the work (not directly to fund the study), technical support etc

Reviewer #2: The data is nearly ten years old on a subject of trauma. Trauma related findings and information shared have no new information. In fact cost is mixed up with injuries in a unimpressive style. Data is taken from insurance companies thus even injury pattern and its impact on the children and families can not be determined. There is unnecessary verbosity in the introduction. References are irrationally used. There are eight references in the discussion section put together in an unintelligent style like 30, 31,32,33 and 35, 36, 37, 38, 39, 40. It appears that authors are not keen interest in comparing with the reported literature. There are huge limitations already identified by the authors too.

**********

6. PLOS authors have the option to publish the peer review history of their article (what does this mean?). If published, this will include your full peer review and any attached files.

If you choose “no”, your identity will remain anonymous but your review may still be made public.

**Do you want your identity to be public for this peer review?**  For information about this choice, including consent withdrawal, please see our Privacy Policy.

Reviewer #1: **Yes:** August Kuwawenaruwa

Reviewer #2: **Yes:** JAMSHED AKHTAR

**********

[NOTE: If reviewer comments were submitted as an attachment file, they will be attached to this email and accessible via the submission site. Please log into your account, locate the manuscript record, and check for the action link "View Attachments". If this link does not appear, there are no attachment files.]

To ensure your figures meet our technical requirements, please review our figure guidelines: https://journals.plos.org/plosone/s/figures

You may also use PLOS’s free figure tool, NAAS, to help you prepare publication quality figures: https://journals.plos.org/plosone/s/figures#loc-tools-for-figure-preparation.

NAAS will assess whether your figures meet our technical requirements by comparing each figure against our figure specifications.

# Author comment: Author response to Decision Letter 1

**DOI:** 10.1371/journal.pone.0353679.r002

11 Jun 2026

Dear Editors and Reviewers,

On behalf of my co-authors, thank you for the careful and constructive reviews of our manuscript, “Incidence and direct medical costs of child injuries in Lebanon (2012–2016): Evidence from closed insurance claims analysis.” We are grateful for the opportunity to revise our submission.

In this revision, we have implemented changes to improve clarity, transparency, and readability. Specifically, we clarified our definition of pediatric patients (<18 years), incorporated age-group categorization in the descriptive presentation, revised statistical significance wording, and reformatted and updated tables (including revised copies of Tables 2 and 3). We also strengthened the Discussion and Conclusion and clarified limitations inherent to closed insurance-claims data, including constrained clinical detail and the inability to measure indirect costs or injury mechanisms.

We provide below a point-by-point response to each comment and indicate where changes were made in the revised manuscript and tables. We appreciate the reviewers’ guidance in improving the manuscript and hope the revised version meets the journal’s standards.

Sincerely,

Dr. Samar Al Hajj

Corresponding Author

American University of Beirut

Email: sh137@aub.edu.lb

When submitting your revision, we need you to address these additional requirements.

1. Please ensure that your manuscript meets PLOS ONE's style requirements, including those for file naming. The PLOS ONE style templates can be found at

https://journals.plos.org/plosone/s/file?id=wjVg/PLOSOne_formatting_sample_main_body.pdf and

https://journals.plos.org/plosone/s/file?id=ba62/PLOSOne_formatting_sample_title_authors_affiliations.pdf

Thank you. We have revised the manuscript, tables and figures to be in the journals requirements.

2. We note that you have indicated that there are restrictions to data sharing for this study. For studies involving human research participant data or other sensitive data, we encourage authors to share de-identified or anonymized data. However, when data cannot be publicly shared for ethical reasons, we allow authors to make their data sets available upon request. For information on unacceptable data access restrictions, please see http://journals.plos.org/plosone/s/data-availability#loc-unacceptable-data-access-restrictions.

Before we proceed with your manuscript, please address the following prompts:

a) If there are ethical or legal restrictions on sharing a de-identified data set, please explain them in detail (e.g., data contain potentially identifying or sensitive patient information, data are owned by a third-party organization, etc.) and who has imposed them (e.g., a Research Ethics Committee or Institutional Review Board, etc.). Please also provide contact information for a data access committee, ethics committee, or other institutional body to which data requests may be sent.

b) If there are no restrictions, please upload the minimal anonymized data set necessary to replicate your study findings to a stable, public repository and provide us with the relevant URLs, DOIs, or accession numbers. Please see http://www.bmj.com/content/340/bmj.c181.long for guidelines on how to de-identify and prepare clinical data for publication. For a list of recommended repositories, please see https://journals.plos.org/plosone/s/recommended-repositories. You also have the option of uploading the data as Supporting Information files, but we would recommend depositing data directly to a data repository if possible.

Please update your Data Availability statement in the submission form accordingly.

Thank you for your comment. Data will be made available upon reasonable request from the corresponding author (sh137@aub.edu.lb). De-identified data cannot be made publicly available as they are acquired from four Third-Party Administrators for health insurance companies, and thus are not owned by the authors’ of the study.

3. Please include a new copy of Tables 2 and 3 in your manuscript; the current table is difficult to read. Please follow the link for more information: https://journals.plos.org/plosone/s/tables

Thank you for your comment. We have modified Tables 2 and 3 to meet the requirements.

4. If the reviewer comments include a recommendation to cite specific previously published works, please review and evaluate these publications to determine whether they are relevant and should be cited. There is no requirement to cite these works unless the editor has indicated otherwise.

Thank you for your comment. Well noted.

Reviewer #1: Abstract

1). A clear definition of a paediatric patient should be articulated, as a paediatric patient is an individual who is a minor, typically defined as being under 15 years of age (and in some jurisdictions, under 18 or 21 years of age).

Thank you for your comment. Please note that within the context of this paper, Paediatric patients are defined as children below the age of 18 (stated In the Method section).

2). The results would benefit from categorisation into specific age groups:

• neonates (birth to 28 days),

• infants (29 days to under 2 years),

• children (2 to under 12 years), and

• adolescents (12 to 18 or 21 years, depending on the authors' definition).

The breakdown of the population represented has been categorized by age group in Table 1. Due to sufficient data in the administrative dataset neonates and infants were combined into one category, children 2 to under 12, and adolescents 12 to under 18 are now listed. Further, we have created a new figure 2 to demonstrate the contributions of injuries to each of these age groups stratified by disposition (ED vs Admitted) to the costs in our results.

3). Cost presentation would also be enhanced by categorizing the data, rather than currently providing a total for all claims, while specific types of injuries are presented as proportions.

Thank you for this suggestion. Cost presentation is categorized in Table 3. This is limited by the nature of the data from the closed insurance claims where the majority of the claims were not categorized and simply billed as a global charge. Particularly for the 86% of cases that were seen and discharged after emergency department treatment

4). The conclusion requires revision; for instance, the results do not provide any information regarding interventions related to pediatric safety and security or indirect costs.

Thank you for your comment. The abstract has been revised to align with your suggestions.

Introduction

5). A clear definition of a pediatric patient should be articulated.

Thank you for your comment. Please note that within the context of this paper, Paediatric patients are defined as children below the age of 18 (stated In the Method section).

6). Authors have cited many articles; for example, on page 3, last paragraph [9, 10, 11, 12, 13, ...], there is a lack of summarized information on what the articles explored and their link to pediatric injury in EMR.

Thank you for your comment. We have retained the most relevant literature that supports the introduction. Some statements are supported by multiple references because there are relatively few studies addressing this topic, and these references collectively provide the necessary context and evidence for the points mentioned.

7). Content in some of the paragraphs keep on repeating, from page 3 last paragraph, page 4 first paragraph and the last paragraph.

Thank you for your comment. We have revised the introduction to remove any repetition.

8). The introduction misses out on the country context regarding existing policies, as well as the safety and security of children aged below 18 years.

Thank you for this comment. We have added contextual information on Lebanon’s policies and child safety environment, noting the lack of a comprehensive national child injury prevention strategy and the limited enforcement of safety regulations.

9). The context of health financing is not clearly articulated, encompassing aspects such as user fees, health insurance, and funding from development partners. Does health insurance cover all the medical costs or patients have to pay some co-payments, for health commodities and admission?

Thank you for this comment. Lebanon has a mixed health financing system that includes public coverage, private health insurance, and out-of-pocket payments, with private insurance commonly involving co-payments for services such as emergency care and hospital admission. This study focuses on direct medical costs derived from private insurance closed claims and therefore reflects insurer-reimbursed costs only. Costs borne directly by patients or covered through other financing mechanisms were not captured and represent a limitation of this analysis. This was mentioned in the discussion, but additional information has been added to provide context in the introduction.

10). Furthermore, the service delivery context requires clarification. Given that some of the results have been presented in relation to various departments, it is necessary to explicitly delineate the service delivery mechanisms.

Thank you for this comment. In Lebanon, pediatric injury care is primarily delivered through hospital-based emergency departments, with more severe cases requiring inpatient admission and specialized services. We have clarified the service delivery context in the introduction to explain how injuries are managed across different departments. The line item costs, when provided, were categorized by two clinicians with trauma care experience. As stated on page 6 - Each charge was grouped into a service category based on the approved procedure or service by trained clinicians (HM, EP).

Methods

11). First paragraph time frame shown is not clear ...... " De-identified, injury- related, closed insurance claims were obtained from four leading Third Party Administrative (TPA) companies for children (<18 years) with International Classification of Diseases (ICD-10) codes corresponding to Injury, poisoning and certain other consequences of external causes (S00-T88) between the period of 01 January 2012 through 31 December 2016 on September 15, 2018...."

Thank for your pointing out this clerical error. It has been corrected

Ethics declaration

12). The tern "Ethics declatarion" needs to be corrected.

Thank you for your comment. This section has been revised.

13). Revise the ethics to align with journal requirement

Thank you for your comment. This section has been revised.

Methods

14). The section lacks information on

i). Currency conversion USD/LBP (Report the dates of the estimated resource quantities and unit costs, plus the currency and year of conversion).

All costs in this dataset were reported in US dollars as is common in Lebanon. No currency conversion was done. The costs were also analyzed as reported over the five years of the study without adjustment for inflation. Since this is analysis of historical data from closed insurance claims we could not estimate the precise date of processing each claim, only the date of the service provided. Furthermore, in the period of study the inflation rate in Lebanon was low experiencing deceleration and finally deflation with the Consumer Price Index varied from a high of 6.58 in 2012 declining each year to a deflationary period in (2015,-3.75%) and (2016, -0.78%). The cumulative average inflation was only 1.82%. There was a fixed conversion rate of 1USD = LBP 1500 during the period of interest.

ii). Clear description of characteristics of the study population (such as age range, demographics, or clinical characteristics)

The study population is outlined in Table 1 and in the methods section

iii). Setting and location - Provide relevant contextual information that may influence findings.

This is a secondary analysis of an insurance dataset. There is no contextual information regarding the settings and location other than all data is reported from hospitals. This has been further clarified in the introduction/

iv). State the perspective(s) adopted by the study and why chosen

Thank you for this comment. We have revised the Methods to explicitly state the study perspective. This analysis adopts a healthcare payer/claims (third-party administrator/insurer) perspective, estimating direct medical costs captured in closed insurance claims (i.e., allowed/approved amounts recorded in the claims database). We chose this perspective because our data source is administrative claims from multiple TPAs, which reliably capture reimbursed direct medical expenditures, and because payer-perspective estimates are directly relevant for budgeting, reimbursement policy, and identifying high-cost injury categories for prevention and quality improvement. We also clarify that indirect costs (e.g., caregiver productivity loss, transportation, out-of-pocket expenditures not captured in claims) are not included, and therefore the estimates should not be interpreted as societal costs.

v). Describe how costs were valued, adjusted etc.

Costs were taken as reported in US Dollars. They are not adjusted for inflation. As indicated above the period of study chosen was that of high price stability (average inflation of 1.8% over 5 years)

vi). Describe any methods for analysing or statistically transforming data, any extrapolation methods etc.

Additional information was added to the methods section to state that given only positive cost and positive skew of the data, we used a log-transformation to appropriately model the data with regression analysis

vii). The insurance claim contains various levels of service delivery, which vary in terms of charges/reimbursements; aggregation of these levels could be misleading.

The majority (76%) of charges were not categorized and simply presented as a global charge. All charges were for hospital-based emergency department or inpatient care where most injury care is provided in Lebanon

viii). Describe any methods used for estimating how the results of the study vary for subgroups.

We have added subgroup analysis by age groups (less than 2 years, 2 to less than 12, 12 to less than 18)

ix). Clear stating the cost comparison being presented in the results section (male vs female, facility level [such as Primary Healthcare, Advanced Specialized Care, Super Specialized Care, etc], health facility departments etc)

The only comparison being made in the results section was between male and female presentations and between Emergency Department treatment vs inpatient hospital treatment costs. All charges were for hospital-based emergency department or inpatient care where most injury care is provided in Lebanon. No data was presented from primary health centers or other non-hospital facilities

15). Data analysis

i). How were patterns assessed and presented in the results section? Also pediatric visits needs clarity in the methods section, for the 2nd or 3rd visits were these linked to the single case of injury or multiple cases of injury?

As stated in the Data Analysis section we only use the first visit per patient for the regression analysis. We also summarize each injury (diagnosis code) at separate visits descriptively

ii). Explain the reason behind decision to present the findings by gender and not by any other categorization (age, health facility level etc)

The convention of presenting the data by sex was maintained. We have added the breakdown of age groups for pediatric injury presentations. All data is from hospital visits only.

iii). A log-transformed generalized liner model was used to analyze costs of adult injury, does this mean claim data was filtered to reflect 12 to 18 or 21 years or all the pediatric patient?

Thank you for pointing out this typo. We have updated to read “cost of pediatric injury” since this data focuses on children, < 18 yr.

iV) A p-value of 0.05 was considered statistically significant. This should be amended to a p-value of less than 0.05 was considered statistically significant.

Thank you. We have changed the wording

v). Use of mean and median: was the claim data normally distributed?

Given only positive cost and positive skew of the data, we used a

Attachment Submitted filename: RTR_Plosone_Final.pdf

# Peer review: Decision Letter 1

**DOI:** 10.1371/journal.pone.0353679.r003

**Authors:** Mabel Aoun

29 Jun 2026

<p>Incidence and direct medical costs of child injuries in Lebanon (2012-2016): Evidence from closed insurance claims analysis

PONE-D-25-26319R1

Dear Dr. Al-Hajj,

We’re pleased to inform you that your manuscript has been judged scientifically suitable for publication and will be formally accepted for publication once it meets all outstanding technical requirements.

Within one week, you’ll receive an e-mail detailing the required amendments. When these have been addressed, you’ll receive a formal acceptance letter and your manuscript will be scheduled for publication.

An invoice will be generated when your article is formally accepted. Please note, if your institution has a publishing partnership with PLOS and your article meets the relevant criteria, all or part of your publication costs will be covered. Please make sure your user information is up-to-date by logging into Editorial Manager at Editorial Manager® and clicking the ‘Update My Information' link at the top of the page. For questions related to billing, please contact billing support.

If your institution or institutions have a press office, please notify them about your upcoming paper to help maximize its impact. If they’ll be preparing press materials, please inform our press team as soon as possible -- no later than 48 hours after receiving the formal acceptance. Your manuscript will remain under strict press embargo until 2 pm Eastern Time on the date of publication. For more information, please contact onepress@plos.org.

Kind regards,

Mabel Aoun, MD, MPH

Academic Editor

PLOS One

Additional Editor Comments (optional):

Reviewers' comments:

Reviewer's Responses to Questions

**Comments to the Author**

1. If the authors have adequately addressed your comments raised in a previous round of review and you feel that this manuscript is now acceptable for publication, you may indicate that here to bypass the “Comments to the Author” section, enter your conflict of interest statement in the “Confidential to Editor” section, and submit your "Accept" recommendation.

Reviewer #2: All comments have been addressed

**********

2. Is the manuscript technically sound, and do the data support the conclusions?

The manuscript must describe a technically sound piece of scientific research with data that supports the conclusions. Experiments must have been conducted rigorously, with appropriate controls, replication, and sample sizes. The conclusions must be drawn appropriately based on the data presented.

Reviewer #2: Yes

**********

3. Has the statistical analysis been performed appropriately and rigorously?

Reviewer #2: I Don't Know

**********

4. Have the authors made all data underlying the findings in their manuscript fully available?

The PLOS Data policy requires authors to make all data underlying the findings described in their manuscript fully available without restriction, with rare exception (please refer to the Data Availability Statement in the manuscript PDF file). The data should be provided as part of the manuscript or its supporting information, or deposited to a public repository. For example, in addition to summary statistics, the data points behind means, medians and variance measures should be available. If there are restrictions on publicly sharing data—e.g. participant privacy or use of data from a third party—those must be specified.

Reviewer #2: No

**********

5. Is the manuscript presented in an intelligible fashion and written in standard English?

PLOS ONE does not copyedit accepted manuscripts, so the language in submitted articles must be clear, correct, and unambiguous. Any typographical or grammatical errors should be corrected at revision, so please note any specific errors here.

Reviewer #2: Yes

**********

6. Review Comments to the Author

Please use the space provided to explain your answers to the questions above. You may also include additional comments for the author, including concerns about dual publication, research ethics, or publication ethics. (Please upload your review as an attachment if it exceeds 20,000 characters)

Reviewer #2: Thank you for responding and clarifying number of issues highlighted during initial review. Though I still data has several limitations in context of injury pattern over a decade as well as cost of the treatment incurred. In context of last few years because political instability, internal migration, and several other factors, the present data fall short of providing true picture and may not be in line with ground realities. However, it did provided a template on which future studies may be conducted.

**********

7. PLOS authors have the option to publish the peer review history of their article (what does this mean?). If published, this will include your full peer review and any attached files.

If you choose “no”, your identity will remain anonymous but your review may still be made public.

**Do you want your identity to be public for this peer review?**  For information about this choice, including consent withdrawal, please see our Privacy Policy.

Reviewer #2: **Yes:** Jamshed Akhtar

**********

# Editor report: Acceptance letter

**DOI:** 10.1371/journal.pone.0353679.r004

**Authors:** Mabel Aoun

PONE-D-25-26319R1

PLOS One

Dear Dr. Al-Hajj,

I'm pleased to inform you that your manuscript has been deemed suitable for publication in PLOS One. Congratulations! Your manuscript is now being handed over to our production team.

At this stage, our production department will prepare your paper for publication. This includes ensuring the following:

* All references, tables, and figures are properly cited

* All relevant supporting information is included in the manuscript submission,

* There are no issues that prevent the paper from being properly typeset

You will receive further instructions from the production team, including instructions on how to review your proof when it is ready. Please keep in mind that we are working through a large volume of accepted articles, so please give us a few days to review your paper and let you know the next and final steps.

Lastly, if your institution or institutions have a press office, please let them know about your upcoming paper now to help maximize its impact. If they'll be preparing press materials, please inform our press team within the next 48 hours. Your manuscript will remain under strict press embargo until 2 pm Eastern Time on the date of publication. For more information, please contact onepress@plos.org.

You will receive an invoice from PLOS for your publication fee after your manuscript has reached the completed accept phase. If you receive an email requesting payment before acceptance or for any other service, this may be a phishing scheme. Learn how to identify phishing emails and protect your accounts at https://explore.plos.org/phishing.

If we can help with anything else, please email us at customercare@plos.org.

Thank you for submitting your work to PLOS ONE and supporting open access.

Kind regards,

PLOS ONE Editorial Office Staff

on behalf of

Pr Mabel Aoun

Academic Editor

PLOS One
